# Mismatch between urban eco-sanitation improvement and health service supply capacity: evidence from Chinese city panel data and interpretable machine learning

**DOI:** 10.1186/s12913-026-15444-8

**Published:** 2026-08-25

**Authors:** Zhen Peng, Lin Wang

**Affiliations:** School of Architecture and Urban Planning, Qingdao University of Technology, Qingdao, 266033 China

**Keywords:** Health service supply capacity, Healthcare resource allocation, Urban eco-sanitation environment, Health service governance, Chinese cities, Interpretable machine learning

## Abstract

**Background:**

Health service supply capacity depends not only on medical resource stocks but also on urban eco-sanitation conditions and resource-allocation mechanisms. Existing studies focus mainly on environmental exposure and health outcomes, with limited attention to whether eco-sanitation improvements can be converted into health service supply capacity.

**Methods:**

This study constructed a panel of Chinese prefecture-level and above cities for 2002–2024, using the health service supply capacity index as the dependent variable and the eco-sanitation environment index as the core explanatory variable. Two-way fixed effects models, dimension-specific analysis, mechanism tests, heterogeneity analysis, robustness checks, and interpretable machine learning identified mismatch mechanisms and boundary conditions. The mechanism analysis was extended to two- and three-year ESE lags and one- and two-year lagged investment variables. Carbon-emission comparability was reassessed on exact CEADs-matched samples for 2002–2019. Hu Huanyong line subgroup models were additionally estimated with city and province-year fixed effects.

**Results:**

The eco-sanitation environment, green infrastructure, and municipal sanitation facilities improved continuously, whereas health service supply capacity increased more slowly. The baseline model does not support a stable positive conversion from eco-sanitation improvement to stronger supply capacity. Dimension-specific results reveal differentiated roles. The positive PPI coefficient must not be read as a pollution benefit; it is consistent with a demand-induced or compensatory resource-allocation response. Neither two- to three-year ESE lags nor one- to two-year lagged investment variables produced a stable urban construction investment transmission chain. The positive CEADs interaction was confirmed only within the 2002–2019 matched sample and therefore represents pre-pandemic evidence. The southeastern Hu-line coefficient attenuated and lost conventional significance after province-year policy shocks were absorbed. Machine learning indicates the relationship is shaped by development stage, population size, fiscal capacity, and nonlinear boundaries.

**Conclusions:**

Eco-sanitation improvement is not a sufficient condition for enhancing health service supply capacity. Healthy city development should integrate eco-sanitation governance, infrastructure investment, workforce allocation, and fiscal arrangements into a unified governance framework. Because HSC is a supply-side proxy based on resource counts, these implications concern resource availability and network density rather than quality of care, realized utilization, accessibility, or overall health-system performance.

**Supplementary Information:**

The online version contains supplementary material available at https://doi.org/10.1186/s12913-026-15444-8.

## Background

Urbanization is reshaping the distribution of population, resources, environmental risks, and public services. Urban agglomeration improves the efficiency of medical care, employment, and infrastructure provision, but it also intensifies air pollution, energy consumption, heat exposure, solid waste, wastewater treatment pressures, and public health service demand. Previous studies have shown that urbanization changes not only health exposure and chronic disease risks, but also healthcare resource allocation, service accessibility, and the structure of public health demand [1–3]. Urban health, therefore, is no longer only an issue within medical institutions; it is the joint outcome of the built environment, ecological infrastructure, municipal sanitation conditions, pollution exposure, fiscal capacity, and the health service system.

Research on high-quality health systems emphasizes that health improvement depends not only on the quantity of medical resources, but also on whether these resources can be effectively organized, made accessible, and converted into real service capacity [4]. Donabedian’s structure-process-outcome framework and the ecology of medical care further indicate that health service capacity should be understood in relation to resource structure, service processes, and the broader social environment [5, 6]. Accordingly, this study defines urban health service supply capacity as the ability of a city, under given population size, eco-sanitation conditions, and public infrastructure constraints, to allocate, support, and continuously provide basic health service resources. The central issue is not the mere stock of medical resources, but whether resources can be effectively allocated and transformed within the urban governance system.

Existing health services research has explained health service capacity from the perspectives of medical utilization, accessibility, and resource allocation [7–9]. At the macro level, the density of the health workforce, primary care systems, and medical service networks is closely related to health system performance [10, 11]. Since China’s health system reform, health resources, insurance coverage, and public health systems have improved continuously, yet resource-allocation disparities remain across regions, urban and rural areas, and institutional tiers [12–14]. Studies on the Healthcare Access and Quality Index and effective coverage index also show that mortality or disease burden alone is insufficient to identify weaknesses in health systems; comprehensive assessment should incorporate supply capacity, service coverage, and population demand [15–17].

At the same time, a growing body of evidence suggests that urban ecological environments and municipal sanitation conditions are important upstream factors shaping health service demand and service capacity. Green space may affect residents’ health by promoting physical activity, relieving psychological stress, reducing heat exposure, mitigating air pollution, and enhancing social interaction [18, 19]. Systematic reviews and meta-analyses have reported significant associations between green-space exposure and all-cause mortality, cardiovascular disease, mental health, pregnancy outcomes, and physiological stress indicators [20–23]. Research on urban green infrastructure further emphasizes that green space is not merely a landscape element, but a joint carrier of ecosystem services, climate adaptation, public health, and social equity [24, 25]. Maas et al. found a stable relationship between green space and residents’ health, while Mitchell and Popham showed that exposure to the natural environment may reduce health inequalities [26, 27]. These studies imply that green infrastructure may not only directly reduce health risks, but also indirectly influence healthcare resource allocation and health service capacity by improving urban livability, governance capacity, and the attractiveness of public services.

Environmental pollution is another key pathway linking the eco-sanitation environment with health service supply capacity. Strong evidence has linked long-term air pollution exposure to mortality risk, cardiopulmonary disease, lung cancer, and loss of life expectancy [28–31]. Global burden of disease studies and the Lancet Commission on pollution and health have shown that air pollution and broader pollution risks cause substantial mortality and disease burden [32–34]. Source-attribution research further indicates that industrial, energy, transport, and residential combustion emissions increase premature mortality through different pathways, while transboundary pollution transport changes the spatial attribution of health damages [35, 36]. In China, evidence from the Huai River policy, national air pollution mapping, and estimates of pollution-related health damages all demonstrate that long-term pollution exposure is closely related to life expectancy losses and health burden [37–40]. Higher pollution pressure may increase latent health service demand and may also crowd out public fiscal and infrastructure resources through environmental governance and disease-burden pressures, thereby weakening the capacity of urban health service systems. Recent Chinese evidence also shows that air pollution can affect migrants’ permanent settlement intentions through subjective attitudes and health status, while ecological, social, infrastructure, and economic resilience are associated with residents’ subjective well-being [41, 42].

In addition to green space and pollution pressure, municipal sanitation infrastructure, such as water supply, wastewater treatment, environmental sanitation, and solid-waste management, provides the basic foundation of urban public health systems. Studies on water, sanitation, and hygiene interventions have shown that improved water and sanitation conditions can substantially reduce diarrhoeal disease risks [43, 44]. Mara et al. and Bartram and Cairncross emphasized that sanitation facilities are not peripheral to traditional medical systems, but constitute a foundational component of public health [45, 46]. Global burden of disease studies have further estimated the health losses attributable to inadequate water, sanitation, and hygiene, while cost-benefit analyses have argued that health benefits should be incorporated into evaluations of water-supply and sanitation infrastructure investments [47–49]. Thus, sewage treatment rates, centralized sewage treatment rates, harmless domestic waste treatment rates, water supply coverage, and gas coverage can be regarded as important components of the urban eco-sanitation environment.

Health service supply capacity is also affected by spatial accessibility and the structure of urban resource allocation. Classic accessibility research argues that access depends not only on whether services exist, but also on the spatial, economic, and organizational match between resources and demand [50]. Luo and Wang systematically examined measures of spatial accessibility to healthcare in a GIS environment, and Wang and Luo further integrated spatial and non-spatial factors to identify health professional shortage areas [51, 52]. Methods such as the enhanced two-step floating catchment area approach have advanced healthcare accessibility research from static supply indicators to spatial supply-demand matching measures [53]. However, fine-grained geographic accessibility and residents’ healthcare-seeking behavior data are not always available in long-term cross-city panel research. Therefore, city-level indicators such as hospital and health-center beds per 10,000 persons, licensed physicians per 10,000 persons, and hospitals and health centers per 10,000 persons cannot fully represent micro-level accessibility, but they still provide long-term, comparable proxies for supply-side health service capacity.

Energy consumption and carbon emissions provide a broader environmental pressure context for understanding the relationship between the eco-sanitation environment and health service supply capacity. City-level carbon-emission accounting in China has developed into a relatively mature framework and can support research on urban low-carbon governance, environmental risks, and public health [54, 55]. Energy consumption, industrial structure, and carbon emissions are often intertwined with pollution exposure, infrastructure efficiency, and urban governance capacity [56, 57]. Therefore, high energy intensity and high carbon emissions may reflect not only resource and environmental pressures associated with urban development patterns, but also changes in the marginal relationship between eco-sanitation improvement and health service supply capacity. In other words, whether eco-sanitation construction can be converted effectively into stronger health service supply capacity may depend on whether a city can simultaneously move beyond development constraints characterized by high pollution, high energy consumption, and high carbon emissions.

In recent years, the combination of panel econometric models and interpretable machine learning has provided a new methodological pathway for urban environmental health research. Random forests and gradient boosting models can capture complex nonlinear relationships and variable importance [58, 59], while interpretability tools such as SHAP and LIME further improve the explainability of machine learning results [60, 61]. However, machine learning mainly supports prediction, identification of variable contributions, and discovery of nonlinear patterns; it cannot replace fixed effects models, lag structures, and robustness checks in identifying average associations and directional relationships. Therefore, in city panel studies, a more appropriate strategy is to use panel econometric models to identify average relationships between the eco-sanitation environment and health service supply capacity, while using interpretable machine learning to supplement the analysis of variable importance, threshold patterns, and potential heterogeneity.

**Fig. 1:**
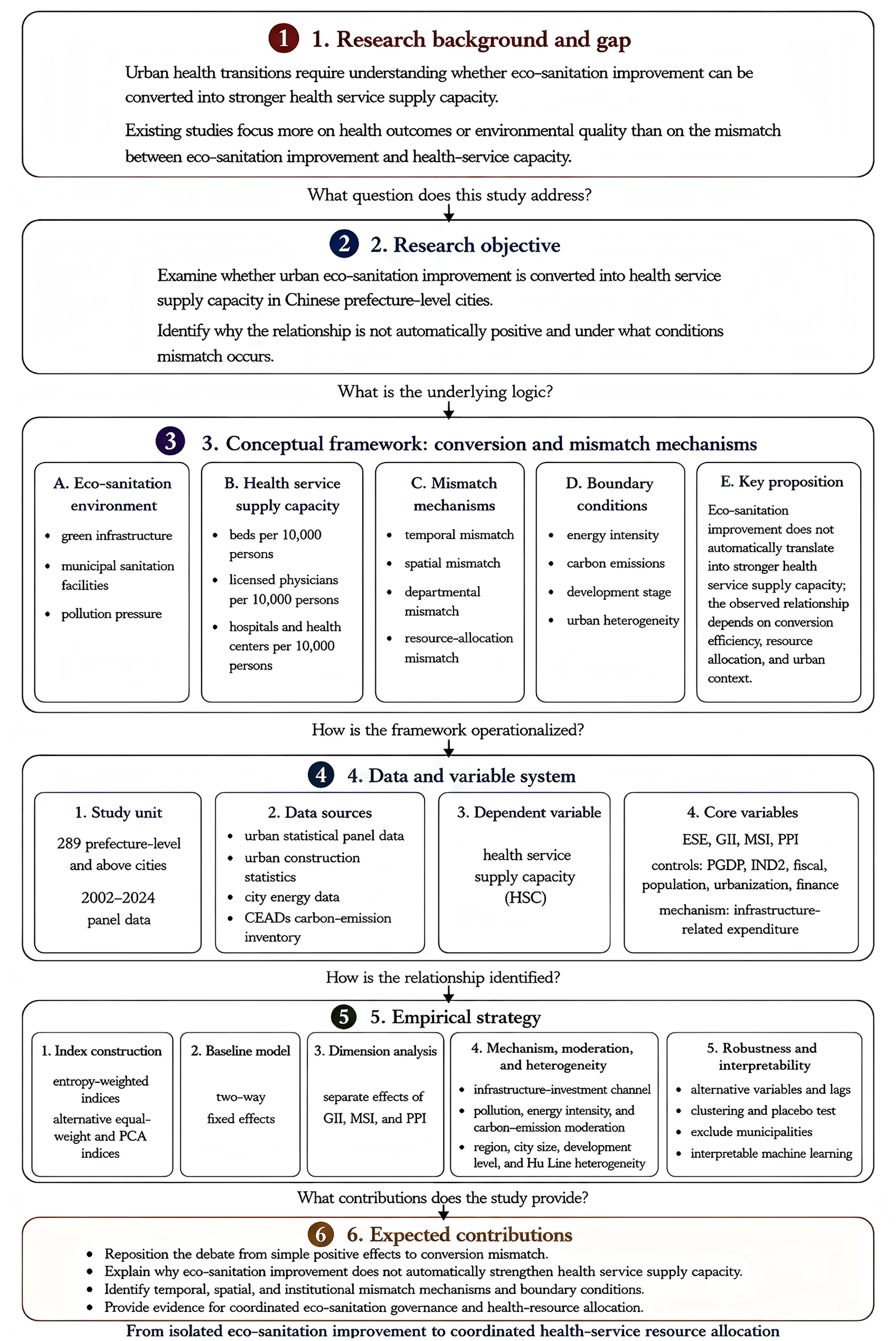
Research framework and methodological route

In summary, three gaps remain in the literature. First, most environmental health studies focus on mortality, disease burden, or individual health outcomes, while relatively few studies examine how the urban eco-sanitation environment affects health service supply capacity and resource allocation from a health services research perspective. Second, green infrastructure, water and gas supply, wastewater treatment, solid-waste management, and pollution pressure are often studied separately, and there is a lack of an integrated analytical framework that connects urban eco-sanitation conditions with the supply capacity of health service systems. Third, few studies of Chinese cities have simultaneously examined whether eco-sanitation improvement can be converted into stronger health service supply capacity and why this conversion may be constrained by energy-carbon conditions, public finance, healthcare resource allocation, and urban heterogeneity. Against this background, this study constructs a city panel dataset for China covering 2002–2024, uses urban health service supply capacity as the dependent variable and the urban eco-sanitation environment as the core explanatory variable, and combines two-way fixed effects models, mechanism tests, heterogeneity analysis, robustness checks, and interpretable machine learning to identify mismatch mechanisms and boundary conditions in the conversion from eco-sanitation improvement to health service supply capacity. Figure 1 presents the research framework and methodological route.

Accordingly, this study focuses on the conversion relationship between eco-sanitation improvement and health service supply capacity and proposes the following research questions: RQ1, can improvements in the urban eco-sanitation environment be stably converted into stronger health service supply capacity? RQ2, do green infrastructure, municipal sanitation facilities, and pollution pressure play differentiated roles in this relationship? RQ3, do urban construction investment and public fiscal capacity constitute effective channels through which the eco-sanitation environment is converted into health service supply capacity? RQ4, do pollution pressure, energy intensity, and carbon emissions modify the marginal relationship between the eco-sanitation environment and health service supply capacity? RQ5, does this relationship exhibit city-type heterogeneity and nonlinear boundaries?

The contributions of this study are threefold. First, it incorporates urban health service supply capacity into the eco-sanitation environment research framework, but does not presuppose an automatic positive conversion between the two; instead, it examines from a health services research perspective whether eco-sanitation improvement can be translated into comparable service supply capacity. Second, it constructs an urban eco-sanitation analytical framework that includes green infrastructure, municipal sanitation facilities, pollution pressure, energy intensity, and carbon emissions, thereby identifying structural mismatches between the eco-sanitation environment and health service supply capacity. Third, by combining two-way fixed effects models with interpretable machine learning, it reveals how such mismatches manifest across time, space, administrative sectors, and stages of urban development, providing empirical evidence for coordinated healthcare resource allocation, primary service network planning, and healthy city governance.

## Methods

### Data sources and sample construction

This study adopts a long-term city-level panel design, taking Chinese prefecture-level and above cities as observational units and city-year observations as the basic analytical unit. It examines the conversion relationship and mismatch mechanisms between eco-sanitation improvement and urban health service supply capacity. The object of analysis is not individual health outcomes or the performance of a single medical institution, but the supply-side capacity of urban health service systems. Therefore, the urban health service supply capacity index (HSC) is used as the dependent variable, and the eco-sanitation environment index (ESE) is used as the core explanatory variable. Considering that eco-sanitation infrastructure construction, green-space improvement, and enhancement of municipal sanitation conditions may affect healthcare resource allocation with a time lag, the baseline model uses the one-year lagged eco-sanitation environment index (ESE_i,t-1).

The data come from four types of city-level statistical sources [62]. The first is a comprehensive urban panel dataset, which provides GDP, population, fiscal revenues and expenditures, industrial structure, health resources, industrial pollution emissions, and PM₂.₅, and is used to construct health service supply capacity, control variables, and pollution pressure indicators. The second is the panel dataset from the China Urban Construction Statistical Yearbook, which provides water supply, gas supply, wastewater treatment, waste treatment, green space, and built-up area indicators, and is the main source for constructing the eco-sanitation environment, green infrastructure, and municipal sanitation facility indices. The third is prefecture-level city energy consumption data, which provide total energy consumption, energy intensity per unit GDP, and per capita energy consumption for energy-constraint and extended analyses. The fourth is the CEADs city-level sectoral carbon-emission inventory, which is used to incorporate the carbon-emission dimension. Because the CEADs series ends in 2019, all carbon-emission moderation results are treated as matched-sample pre-pandemic extensions and are not extrapolated to 2020–2024. The data sources and uses are shown in Table 1.

**Table 1:** Data sources and uses

Data source	Year range	Main variables	Use in this study
Comprehensive urban panel data	2000–2024	GDP, population, industrial structure, fiscal revenues and expenditures, health resources, industrial pollution emissions, PM₂.₅	Construction of health service supply capacity, control variables, and pollution pressure indicators
China Urban Construction Statistical Yearbook panel	2002–2024	Water supply, gas supply, sewage treatment, waste treatment, green space, and built-up area indicators	Construction of eco-sanitation environment, green infrastructure, and municipal sanitation facility indices
Prefecture-level city energy consumption data	2006–2024	Total energy consumption, energy intensity per unit GDP, and per capita energy consumption	Energy constraints, moderation analysis, and extended analysis
CEADs city-level sectoral carbon-emission inventory	1997–2019	City-level sectoral CO₂ emissions	Carbon-emission moderation and extended analyses restricted to successfully matched observations through 2019; no post-2019 extrapolation

The sample was constructed by merging data using city administrative division codes and year. For CEADs data without unified city codes, province and city names were first standardized and then matched to administrative division codes through city-name aliases. Observations with multiple candidates, unidentifiable administrative boundary changes, or unstable city-name matches were excluded from carbon-emission extended models. For cities that experienced administrative boundary adjustments, name changes, or code changes during the study period, the analysis used continuously traceable prefecture-level city administrative codes as the baseline. City-year observations that could not be stably traced to the same administrative unit were excluded from the baseline model to ensure consistency in spatial units.

The main sample covers 2002–2024 and includes 289 cities and 6,647 city-year observations. The baseline model requires non-missing values for HSC, one-year lagged ESE, and the main controls, resulting in 5,070 usable observations. The energy-extended sample covers 2006–2024 and includes 279 cities and 5,301 observations, of which 4,178 satisfy the extended-model conditions. The CEADs extended sample matches 257 cities and covers 2002–2019 only. The main_ceads_2002_2019 sample contains 5,202 observations, of which 3,147 are model-usable; the energy_ceads_2006_2019 sample contains 3,906 observations, of which 2,748 are model-usable. This temporal boundary means that CEADs-based estimates cannot capture pandemic-related or post-2019 changes in healthcare demand, public-health expenditure, hospital capacity, or intergovernmental resource allocation. The sample structure is shown in Table 2.

**Table 2:** Sample structure

Sample	Data sheet	Year range	Cities	Observations	Model-usable observations	Main use
Main sample	main_2002_2024	2002–2024	289	6647	5070	Baseline two-way fixed effects model
Energy-extended sample	energy_2006_2024	2006–2024	279	5301	4178	Models including energy consumption, energy intensity, and per capita energy consumption
CEADs extended sample	main_ceads_2002_2019	2002–2019	257	5202	3147	Models including city-level sectoral carbon emissions; interpreted as pre-pandemic evidence only
Energy-CEADs extended sample	energy_ceads_2006_2019	2006–2019	257	3906	2748	Models including both energy variables and carbon emissions; interpreted as pre-pandemic evidence only

Data preprocessing included standardizing city names, city codes, and year formats; removing duplicate city-year records; harmonizing variable units and statistical definitions; and winsorizing continuous indicators at the 1st and 99th percentiles. Derived variables were further constructed, including health resources per 10,000 persons, pollution emissions per unit GDP, fiscal expenditure as a share of GDP, fixed-asset investment as a share of GDP, loan balance as a share of GDP, and the logarithm of per capita GDP. The baseline estimation uses complete-case samples and does not impute core variables. For monetary variables such as GDP, fiscal expenditure, fixed-asset investment, loan balance, and urban construction investment, this study preferentially uses ratios to GDP or logarithmic forms; where cross-year absolute monetary comparisons are involved, values are converted to comparable prices using the corresponding price indices. In the mechanism tests, urban maintenance and construction expenditure, landscaping investment, wastewater treatment investment, sanitation and environmental appearance investment, and garbage-treatment investment are all converted to shares of GDP, while year fixed effects further absorb national price changes and common macroeconomic trends.

### Variable definitions

The dependent variable is the urban health service supply capacity index (HSC_it), which measures a city’s ability to allocate and provide basic health service resources for a given population size. The index consists of three positive indicators: hospital and health-center beds per 10,000 persons (health_beds_per_10k), licensed assistant and practicing physicians per 10,000 persons (licensed_doctors_per_10k), and hospitals and health centers per 10,000 persons (hospitals_health_centers_per_10k). These indicators reflect medical facility capacity, health workforce supply, and service network density, respectively.

It should be noted that HSC is operationalized as a supply-side proxy index. Although the three component indicators are standardized per 10,000 persons and therefore incorporate population size, they primarily measure the stock of beds and physicians and the density of hospitals and health centers. They do not adjust for clinical quality, staffing mix, equipment capability, service efficiency, actual utilization, unmet need, spatial accessibility, patient experience, or health outcomes. Accordingly, HSC is interpreted throughout as a comparable city-level measure of resource availability and service-network density, not as a complete measure of effective coverage or health-system performance.

The core explanatory variable is the one-year lagged eco-sanitation environment index (ESE_i,t-1). ESE_it is composed of park green area per capita, built-up area green coverage rate, built-up area green-space rate, water supply coverage, gas coverage, sewage treatment rate, centralized sewage treatment rate, and harmless domestic waste treatment rate. It comprehensively reflects urban green space, municipal sanitation facilities, and basic eco-sanitation conditions. The baseline model uses lagged ESE to reduce the risk of reverse association between contemporaneous expansion of health services and eco-sanitation construction.

To identify the internal structure of the eco-sanitation environment, this study further constructs a green infrastructure index (GII), a municipal sanitation facility index (MSI), and a pollution pressure index (PPI). GII_it is composed of park green area per capita, built-up area green coverage rate, and built-up area green-space rate. MSI_it is composed of water supply coverage, gas coverage, sewage treatment rate, centralized sewage treatment rate, and harmless domestic waste treatment rate. PPI_it is composed of industrial wastewater, sulfur dioxide, smoke and dust, and nitrogen oxide emissions per unit GDP, together with annual mean PM₂.₅ concentration. A higher PPI indicates greater pollution exposure and governance pressure.

The control vector X_it is included to reduce omitted-variable bias arising from differences in urban economic development, industrial structure, fiscal capacity, population size, urbanization, investment, and financial conditions. The baseline model includes the logarithm of per capita GDP (ln_pgdp_yuan), the share of secondary industry in GDP (secondary_industry_share), and local fiscal expenditure as a share of GDP (fiscal_expenditure_gdp). Extended models further include the tertiary industry share, population size, urbanization rate, population density, fixed-asset investment, loan balance, education expenditure, science and technology expenditure, and R&D intensity. Energy-extended analyses use total energy consumption, energy intensity per unit GDP, and per capita energy consumption. Carbon-emission extended analyses use CEADs city-level sectoral CO₂ emissions. Mechanism tests use urban maintenance and construction expenditure, landscaping investment, sanitation and environmental appearance investment, wastewater treatment investment, garbage-treatment investment, and local fiscal expenditure/GDP as a broader fiscal-capacity indicator. Heterogeneity analyses use grouping variables for region, the Hu Huanyong line, city size, economic development level, and fiscal capacity. The main variable definitions are shown in Table 3.

**Table 3:** Variable definitions and model roles

Category	Symbol	Variable name	Measurement	Model role
Dependent variable	*HSC*_*it*_	health_capacity_index	Entropy-weighted index based on beds, licensed physicians, and hospitals/health centers per 10,000 persons	Measures urban health service supply capacity
Core explanatory variable	*ESE*_*i,t-1*_	eco_sanitation_index_l1	One-year lagged eco-sanitation environment index including green space, water supply, gas supply, sewage treatment, and waste treatment indicators	Core variable in the baseline model
Green infrastructure	*GII*_*i,t-1*_	green_infrastructure_index_l1	Park green area per capita, built-up area green coverage rate, and built-up area green-space rate	Dimension-specific test
Municipal sanitation facilities	*MSI*_*i,t-1*_	municipal_sanitation_index_l1	Water supply coverage, gas coverage, sewage treatment rate, centralized sewage treatment rate, and harmless domestic waste treatment rate	Dimension-specific test
Pollution pressure	*PPI*_*i,t-1*_	pollution_pressure_index_l1	Industrial wastewater, SO₂, smoke/dust, and NOₓ emissions per unit GDP, plus PM₂.₅; higher values indicate greater pollution pressure	Control variable, dimension-specific variable, or moderator
Energy variables	*Energy*_*i,t-1*_	energy_intensity_10k_tce_per_100m_yuan_l1, etc.	Total energy consumption, energy intensity per unit GDP, and per capita energy consumption	Energy-extended model
Carbon-emission variable	*CO*_*2it*_	ceads_sector_co2_million_ton	CEADs city-level sectoral CO₂ emissions, measured in million tons	Carbon-emission extended model
Control variables	*X*_*it*_	ln_pgdp_yuan, secondary_industry_share, fiscal_expenditure_gdp, etc.	Economic development, industrial structure, fiscal capacity, population size, urbanization, investment, and financial conditions	Reduce omitted-variable bias
Mechanism variables	*M*_*it*_	Variables related to construction, green space, sanitation, sewage, and fiscal capacity	Urban maintenance and construction expenditure, landscaping investment, sanitation and environmental appearance investment, wastewater treatment investment, garbage-treatment investment, and local fiscal expenditure/GDP as a broader fiscal-capacity indicator	Mechanism test
Grouping variables	*G*_*i*_	region, city_size, huhuan_yong, pgdp_group, fiscal_group, etc.	Region, city size, Hu Huanyong line, economic development level, and fiscal capacity	Heterogeneity analysis

### Index construction method

This study uses the entropy weighting method to construct composite indices. The basic logic is that if an indicator varies more across city-year observations, it contains more information and receives a larger weight in the composite index; if an indicator varies less, it receives a lower weight. To ensure that the indices are comparable across years, all composite indices are weighted using the pooled city-year sample rather than being weighted separately by year.

Let *x*_*ijt*_ denote the *j*_th_ raw indicator for city *i* in year *t*. For positive indicators, values are first winsorized at the 1st and 99th percentiles and then standardized using min-max normalization:

Formula (1): Min-max normalization

1 $${z_{ijt}}{\rm{ = }}{{{x_{ijt}}{\rm{ - min}}\left( {{x_j}} \right)} \over {{\rm{max}}\left( {{x_j}} \right){\rm{ - min}}\left( {{x_j}} \right)}}$$

where *z*_*ijt*_ is the standardized indicator value, *x*_*ijt*_ is the raw indicator value, and min(*x*_*j*_) and max(*x*_*j*_) are the minimum and maximum values of indicator *j* in the full city-year sample, respectively. For pollution-pressure indicators, this study focuses on the level of pollution pressure itself; therefore, pollution emissions per unit GDP and PM₂.₅ concentration are also treated as positive pressure indicators, meaning that higher values indicate greater pollution pressure.

After standardization, the share of indicator j in observation it is calculated as follows:

Formula (2): Indicator share

2 $${p_{ijt}}{\rm{ = }}{{{z_{ijt}}{\rm{ + }}\varepsilon } \over {\mathop \sum \nolimits_i \mathop \sum \nolimits_t \left( {{z_{ijt}}{\rm{ + }}\varepsilon } \right)}}$$

where *ε* is a very small positive number used to avoid undefined logarithms when values are zero. The information entropy of each indicator is calculated as follows:

Formula (3): Information entropy

3 $${e_j}{\rm{ = - }}{{\rm{1}} \over {{\rm{ln}}\left( {{n_j}} \right)}}\mathop \sum \limits_i \mathop \sum \limits_t {p_{ijt}}{\rm{ln}}\left( {{p_{ijt}}} \right)$$

where *n*_*j*_ denotes the number of non-missing observations for indicator *j*. The information utility and indicator weight are then calculated as follows:

Formula (4): Information utility

4 $${d_j}{\rm{ = 1 - }}{e_j}$$

Formula (5): Entropy weight

5 $${w_j}{\rm{ = }}{{{d_j}} \over {\mathop \sum \nolimits_j {d_j}}}$$

where *d*_*j*_ denotes the information utility value of indicator *j*, and *w*_*j*_ denotes its entropy weight. The final composite index is calculated as follows:

Formula (6): Composite index

6 $${\rm{Inde}}{{\rm{x}}_{it}}{\rm{ = }}{{\mathop \sum \nolimits_j {w_j}{z_{ijt}}} \over {\mathop \sum \nolimits_j {w_j}I\left( {{z_{ijt}}{\rm{is\: nonmissing}}} \right)}}$$

where *I*(·) is an indicator function. When a city-year observation has some missing component indicators, the index is recalculated by normalizing over the weights of the indicators actually available for that observation. To ensure stable index interpretation, the health service supply capacity index requires at least two valid components, the green infrastructure index requires at least two valid components, the municipal sanitation facility index requires at least three valid components, the eco-sanitation environment index requires at least four valid components, and the pollution pressure index requires at least two valid components. Observations below the minimum number of valid components are set to missing. The components and entropy weights of each composite index are shown in Table 4.

**Table 4:** Components and entropy weights of composite indices

Index system	Original indicator	Variable name	Entropy weight
Health service supply capacity index (HSC)	Hospital and health-center beds per 10,000 persons	health_beds_per_10k	0.2626
Health service supply capacity index (HSC)	Licensed physicians per 10,000 persons	licensed_doctors_per_10k	0.3209
Health service supply capacity index (HSC)	Hospitals and health centers per 10,000 persons	hospitals_health_centers_per_10k	0.4165
Green infrastructure index (GII)	Park green area per capita	park_green_area_pc	0.5754
Green infrastructure index (GII)	Built-up area green coverage rate	builtup_green_coverage_rate	0.2106
Green infrastructure index (GII)	Built-up area green-space rate	builtup_green_space_rate	0.2140
Municipal sanitation facility index (MSI)	Water supply coverage rate	water_supply_coverage_rate	0.1136
Municipal sanitation facility index (MSI)	Gas coverage rate	gas_coverage_rate	0.2038
Municipal sanitation facility index (MSI)	Sewage treatment rate	sewage_treatment_rate	0.2457
Municipal sanitation facility index (MSI)	Centralized sewage treatment rate	centralized_sewage_treatment_rate	0.3085
Municipal sanitation facility index (MSI)	Harmless domestic waste treatment rate	harmless_garbage_treatment_rate	0.1284
Eco-sanitation environment index (ESE)	Park green area per capita	park_green_area_pc	0.2757
Eco-sanitation environment index (ESE)	Built-up area green coverage rate	builtup_green_coverage_rate	0.1009
Eco-sanitation environment index (ESE)	Built-up area green-space rate	builtup_green_space_rate	0.1025
Eco-sanitation environment index (ESE)	Water supply coverage rate	water_supply_coverage_rate	0.0592
Eco-sanitation environment index (ESE)	Gas coverage rate	gas_coverage_rate	0.1062
Eco-sanitation environment index (ESE)	Sewage treatment rate	sewage_treatment_rate	0.1280
Eco-sanitation environment index (ESE)	Centralized sewage treatment rate	centralized_sewage_treatment_rate	0.1607
Eco-sanitation environment index (ESE)	Harmless domestic waste treatment rate	harmless_garbage_treatment_rate	0.0669
Pollution pressure index (PPI)	Industrial wastewater emissions per unit GDP	industrial_wastewater_per_gdp	0.1892
Pollution pressure index (PPI)	Industrial sulfur dioxide emissions per unit GDP	industrial_so2_per_gdp	0.2970
Pollution pressure index (PPI)	Industrial smoke/dust emissions per unit GDP	industrial_smoke_dust_per_gdp	0.2625
Pollution pressure index (PPI)	Industrial nitrogen oxide emissions per unit GDP	industrial_nox_per_gdp	0.1913
Pollution pressure index (PPI)	Annual mean PM₂.₅ concentration	pm25_ug_m3	0.0600

### Econometric model specification

This study first estimates a city and year two-way fixed effects model. The baseline model is specified as follows:

Formula (7): Baseline two-way fixed effects model

7 $${\rm{HS}}{{\rm{C}}_{it}}{\rm{ = }}\alpha {\rm{ + }}\beta \,{\rm{ES}}{{\rm{E}}_{i,t - 1}}{\rm{ + }}{\gamma ^\prime }{X_{it}}{\rm{ + }}{\mu _i} + {\lambda _t} + {\varepsilon _{it}}$$

where *HSC*_*it*_ denotes the health service supply capacity of city *i* in year *t*, *ESE*_*i,t-1*_ denotes the one-year lagged eco-sanitation environment index, *X*_*it*_ is the vector of control variables, *μ*_*i*_ denotes city fixed effects, *λ*_*t*_ denotes year fixed effects, and *ε*_*it*_ denotes the random error term. *β* is the coefficient of primary interest, indicating whether health service supply capacity changes within the same city after eco-sanitation improvement, that is, whether eco-sanitation improvement can be converted into stronger health service supply capacity. Standard errors in all baseline regressions are clustered at the city level to allow serial correlation and heteroskedasticity within cities.

To distinguish the roles of different dimensions of the eco-sanitation environment, this study further decomposes the eco-sanitation environment into green infrastructure, municipal sanitation facilities, and pollution pressure, and estimates the following dimension-specific model:

Formula (8): Dimension-specific model

8 $$\eqalign{{\rm{HS}}{{\rm{C}}_{it}}& {\rm{ = }}\alpha {\rm{ + }}{\beta _{\rm{1}}}\,{\rm{GI}}{{\rm{I}}_{i,t - 1}}{\rm{ + }}{\beta _{\rm{2}}}\,{\rm{MS}}{{\rm{I}}_{i,t - 1}}{\rm{ + }}{\beta _{\rm{3}}}\,{\rm{PP}}{{\rm{I}}_{i,t - 1}} \cr & {\rm{ + }}{\gamma ^\prime }{X_{it}} + {\mu _i} + {\lambda _t} + {\varepsilon _it} \cr} $$

where *GII*_*i,t-1*_, *MSI*_*i,t-1*_, and *PPI*_*i,t-1*_ denote the one-year lagged green infrastructure index, municipal sanitation facility index, and pollution pressure index, respectively. This model examines whether different dimensions of the eco-sanitation environment play different roles in changes in health service supply capacity and identifies whether green space, municipal sanitation infrastructure, and pollution pressure constitute different sources of conversion mismatch.

Pollution pressure, energy intensity, and carbon emissions may modify the marginal effect of eco-sanitation improvement. To test this moderation effect, the following model is specified:

Formula (9): Moderation model

9 $$\eqalign{{\rm{HS}}{{\rm{C}}_{it}} & = \alpha + \beta \,{\rm{ES}}{{\rm{E}}_{i,t - 1}} + \delta {Z_{i,t - 1}} \cr & + \theta \left( {{\rm{ES}}{{\rm{E}}_{i,t - 1}} \times {Z_{i,t - 1}}} \right) + {\gamma ^\prime }{X_{it}} \cr & + {\mu _i} + {\lambda _t} + {\varepsilon _{it}} \cr} $$

where Zi,t-1 sequentially denotes the pollution pressure index, energy intensity per unit GDP, per capita energy consumption, or CEADs city-level sectoral carbon emissions. If the interaction term θ is significant, pollution, energy, or carbon-emission conditions modify the marginal relationship between ESE and HSC; the sign of θ is used to distinguish constraint, compensation, or resource-allocation responses. Continuous interacting variables are mean-centered to reduce multicollinearity. For CEADs, the moderation model is estimated only on observations available through 2019, and an exact-sample baseline comparison is reported so that temporal and sample-composition effects can be distinguished.

The mechanism tests distinguish five project-specific urban public infrastructure investment channels (urban maintenance and construction expenditure, landscaping investment, wastewater treatment investment, sanitation and environmental appearance investment, and garbage-treatment investment) from the broader fiscal-capacity indicator of local fiscal expenditure/GDP. Pollution pressure, energy intensity, and carbon emissions are no longer treated as mechanism variables; instead, their boundary roles are examined in the moderation model described above. The first-stage model for mechanism variable M_it is specified as follows:

Formula (10): First-stage mechanism model

10 $${M_{it}} = \alpha + \rho \,{\rm{ES}}{{\rm{E}}_{i,t - 1}} + {\gamma ^\prime }{X_{it}} + {\mu _i} + {\lambda _t} + {\varepsilon _{it}}$$

In Formula (10), Mit denotes the mechanism variable of city i in year t, and ρ captures the association between lagged ESE and the mechanism variable; the remaining terms are defined as in Formula (7). The original one-year mechanism analysis includes five project-specific investment variables: urban maintenance and construction expenditure/GDP, landscaping investment/GDP, wastewater treatment investment/GDP, sanitation and environmental appearance investment/GDP, and garbage-treatment investment/GDP. Local fiscal expenditure/GDP is included separately as a broader fiscal-capacity indicator. To reflect the multi-year cycle of infrastructure implementation and health-resource allocation, the longer-lag extension is applied to the five project-specific investment variables: the first stage is re-estimated using ESE lags of two and three years, and the outcome stage replaces contemporaneous investment variables with one- and two-year lags. Local fiscal expenditure/GDP is retained only in the original one-year specification and is not included in the extended Table 7 because it represents broad fiscal capacity rather than a project-specific investment flow.

Formula (11): Model with mechanism variable

11 $$\eqalign{{\rm{HS}}{{\rm{C}}_{it}} & = \alpha + \beta \,{\rm{ES}}{{\rm{E}}_{i,t - 1}} + \phi {M_{it}} \cr & + {\gamma ^\prime }{X_{it}} + {\mu _i} + {\lambda _t} + {\varepsilon _{it}} \cr} $$

where Mit denotes the mechanism variable. A possible transmission channel requires a statistically supported ESE-to-mechanism association, a supported mechanism-to-HSC association, and a corresponding change in the ESE coefficient after the mechanism is included. The same criteria are applied to the two- and three-year ESE specifications and to one- and two-year lagged investment variables. Because the study uses observational city-level data, these tests identify potential pathways rather than strict causal mediation.

### Heterogeneity analysis

To examine whether the conversion relationship between the eco-sanitation environment and health service supply capacity varies across urban conditions, this study conducts four types of heterogeneity analysis. First, cities are grouped into eastern, central, western, and northeastern regions to compare differences across regions with different stages of development and fiscal capacity. Second, cities are grouped by their location on either side of the Hu Huanyong line to examine how population density, resource endowments, and urban-system differences affect the estimates. Third, cities are grouped by city size or population size to test whether large and small-to-medium cities differ in the agglomeration of health service resources and the conversion efficiency of eco-sanitation infrastructure. Fourth, cities are divided into high and low groups according to the median of per capita GDP or fiscal expenditure as a share of GDP to examine whether economic development and fiscal capacity affect the conversion of eco-sanitation improvement into health service supply capacity.

Heterogeneity is assessed using subgroup and interaction-term regressions. The primary subgroup models retain city and year fixed effects and the baseline controls. For the Hu Huanyong line comparison, a stricter specification additionally includes province-year fixed effects. Ordinary province indicators are time invariant and nested within city fixed effects; province-year fixed effects are therefore used to absorb time-varying provincial health, fiscal, environmental, and pandemic-response policies that may correlate with the geographic divide.

Formula (12): Heterogeneity interaction model

12 $$\eqalign{{\rm{HS}}{{\rm{C}}_{it}} & = \alpha + \beta \,{\rm{ES}}{{\rm{E}}_{i,t - 1}} + \eta ({\rm{ES}}{{\rm{E}}_{i,t - 1}} \times {G_i}) \cr & + {\gamma ^\prime }{X_{it}} + {\mu _i} + {\lambda _t} + {\varepsilon _{it}} \cr} $$

where Gi denotes the grouping variable, including region, Hu Huanyong line grouping, city size, economic development level, or fiscal capacity. η reflects whether the ESE-HSC relationship varies by city type. In the Hu-line subgroup sensitivity models, province-year fixed effects replace the common year fixed effects because the former already absorb province-specific and national annual shocks.

### Robustness checks and endogeneity treatment

This study evaluates robustness through alternative dependent and explanatory variables, sample restrictions, control sets, weighting methods, and error structures. Lag sensitivity includes contemporaneous ESE, two- and three-year ESE lags, and one- and two-year lagged investment variables. CEADs sensitivity compares a baseline and moderation model on the exact 2002–2019 CEADs sample and a baseline model for the same matched cities over 2002–2019. The Hu Huanyong line subgroup models are re-estimated with city and province-year fixed effects. Additional checks exclude municipalities, vary controls, use city-clustered and city-year two-way clustered standard errors, and replace the entropy-weighted index with equal-weighted and PCA indices.

With respect to endogeneity, this study faces three main risks: reverse causality, omitted variables, and common trends. To mitigate reverse causality, the baseline model uses the one-year lagged eco-sanitation environment index. To reduce omitted-variable bias, the models include city fixed effects, year fixed effects, and multidimensional controls. To address common trends, year fixed effects absorb national shocks, and heterogeneity and sample-adjustment analyses test whether the results are driven by specific regions or years. In addition, this study includes future ESE as a placebo variable and conducts a joint test using one-year, two-year, and three-year leads of ESE to identify possible systematic pre-trends. If the multi-period lead terms are jointly insignificant, current changes in health service supply capacity are unlikely to be driven entirely by future eco-sanitation changes or pre-existing trends; if weak significance is present, the baseline results should be interpreted as directional evidence rather than strict causal effects. The placebo model is specified as follows. It should be emphasized that this study is not based on an exogenous policy shock or a natural experimental design. Fixed effects, lagged variables, and placebo tests can only partially mitigate endogeneity concerns. Therefore, the baseline results are interpreted mainly as conditional within-city associations and directional evidence between changes in the eco-sanitation environment and changes in health service supply capacity, rather than as strict causal effects.

Formula (13): Future-period placebo test model

13 $$\eqalign{{\rm{HS}}{{\rm{C}}_{it}} & = \alpha + {\beta _f}\,{\rm{ES}}{{\rm{E}}_{i,t + 1}} + {\gamma ^\prime }{X_{it}} \cr & + {\mu _i} + {\lambda _t} + {\varepsilon _{it}} \cr} $$

where *ESE*_*i,t+1*_ denotes the future one-year eco-sanitation environment index, and *β*_*f*_ is the placebo coefficient. If *β*_*f*_ is insignificant, the baseline result is unlikely to be fully driven by reverse causality or pre-trends.

### Interpretable machine learning as a supplementary analysis

In addition to panel econometric models, this study uses interpretable machine learning as a supplementary analysis. The machine learning models take the urban health service supply capacity index as the prediction target and use the eco-sanitation environment index, green infrastructure index, municipal sanitation facility index, pollution pressure index, energy intensity, carbon emissions, and control variables as features. The models include random forest and gradient boosting, and prediction performance is reported using R^2^, RMSE, and MAE. To avoid temporal leakage, observations from 2018 and earlier are used as the training set, and observations from 2019 onward are used as the test set, rather than using a completely random split. Hyperparameters are selected through time-blocked cross-validation and fixed in the final reproducible configuration: for the random forest, n_estimators = 500, max_depth = None, max_features = sqrt, and min_samples_leaf = 2; for the gradient boosting model, n_estimators = 300, max_depth = 3, learning_rate = 0.05, subsample = 0.80, and max_features = sqrt. Time-blocked cross-validation uses rolling year-based validation within the training period to avoid information leakage from adjacent years of the same city.

The machine learning analysis has three purposes. First, it compares the relative contributions of eco-sanitation variables with economic, fiscal, and population variables. Second, it identifies nonlinear relationships and potential thresholds, such as whether the marginal contribution of the eco-sanitation environment index, sewage treatment rate, built-up area green coverage rate, or harmless domestic waste treatment rate strengthens or weakens after reaching a certain level. Third, SHAP values are used to explain the direction and magnitude of variable contributions to predictions, helping assess whether average effects in panel models conceal city heterogeneity. It should be emphasized that machine learning results are used only for pattern recognition and interpretive supplementation; because they cannot automatically control for unobserved city heterogeneity or reverse causality, they are not used as the main basis for causal inference.

The predictive performance of the machine learning models is evaluated using the following metrics:

Formula (14): Coefficient of determination R^2^

14 $${R^{\rm{2}}}{\rm{ = 1 - }}{{\mathop \sum \nolimits_{i{\rm{,}}t} {{\left( {{\rm{HS}}{{\rm{C}}_{it}}{\rm{ - }}{{\widehat {{\rm{HSC}}}}_{it}}} \right)}^{\rm{2}}}} \over {\mathop \sum \nolimits_{i{\rm{,}}t} {{\left( {{\rm{HS}}{{\rm{C}}_{it}}{\rm{ - }}\overline {{\rm{HSC}}} } \right)}^{\rm{2}}}}}$$

Formula (15): Root mean squared error (RMSE)

15 $${\rm{RMSE = }}\sqrt {{{\rm{1}} \over N}\sum\nolimits_{i,t} {{{\left( {{\rm{HS}}{{\rm{C}}_{it}}{\rm{ - }}{{\widehat {{\rm{HSC}}}}_{it}}} \right)}^{\rm{2}}}} } $$

Formula (16): Mean absolute error (MAE)

16 $${\rm{MAE = }}{{\rm{1}} \over N}\sum\nolimits_{i,t} {\left| {{\rm{HS}}{{\rm{C}}_{it}}{\rm{ - }}{{\widehat {{\rm{HSC}}}}_{it}}} \right|} $$

where *HSC*_*it*_ denotes the observed health service supply capacity, *ĤSC*_*it*_ denotes the value predicted by the machine learning model, *HS* denotes the sample mean of *HSC*, and *N* denotes the number of observations in the test sample.

## Results

### Descriptive statistics and index evolution

Table 5 reports descriptive statistics for the main variables in the model sample. The mean value of the urban health service supply capacity index is 0.2201, with a standard deviation of 0.1236, indicating substantial differences in health resource supply capacity across cities. The mean eco-sanitation environment index is 0.6419, which is much higher than the health service supply capacity index, suggesting that urban eco-sanitation infrastructure improved relatively rapidly but was not fully synchronized with healthcare resource allocation. The mean green infrastructure index is 0.4860, lower than the municipal sanitation facility index of 0.8048, indicating that water supply, gas coverage, sewage treatment, and waste treatment infrastructure had relatively high coverage, whereas green-space provision still showed larger inter-city variation. The mean pollution pressure index is 0.1442, and its maximum reaches 1, indicating that some cities still faced strong environmental pressure.

**Table 5:** Descriptive statistics of main variables

Variable	N	Mean	SD	Min	Median	Max
Health service supply capacity	5070	0.2201	0.1236	0.0084	0.1987	0.7892
Eco-sanitation environment	5068	0.6419	0.1644	0.0071	0.6853	0.9663
Green infrastructure	5062	0.4860	0.1543	0.0000	0.4968	0.9636
Municipal sanitation facilities	4879	0.8048	0.1860	0.0400	0.8710	1.0000
Pollution pressure	4941	0.1442	0.1442	0.0000	0.0966	1.0000
Hospital and health-center beds per 10,000 persons	5069	39.5731	18.1379	8.6000	37.0235	136.9930
Licensed physicians per 10,000 persons	5069	21.0893	11.4158	3.7554	18.7942	98.3317
Hospitals and health centers per 10,000 persons	5070	0.5205	0.6512	0.0543	0.4068	11.4770
Log per capita GDP	5070	10.2887	0.8425	7.6009	10.3590	12.4565
Secondary industry share	5070	46.6420	11.3195	10.6000	46.9600	90.9700
Fiscal expenditure/GDP	5070	0.1701	0.0998	0.0313	0.1455	1.1817

To examine the external validity of the HSC indicator, this study further tests the correlation between HSC and the number of all health institutions per 10,000 persons, which is not included in the index construction. The Pearson correlation coefficient is 0.379 and the Spearman rank correlation coefficient is 0.495, indicating a moderate positive correlation between HSC and urban health service network density. However, this test provides only supply-side external validity evidence and cannot substitute for indicators of quality of care, healthcare accessibility, or service utilization.

**Fig. 2:**
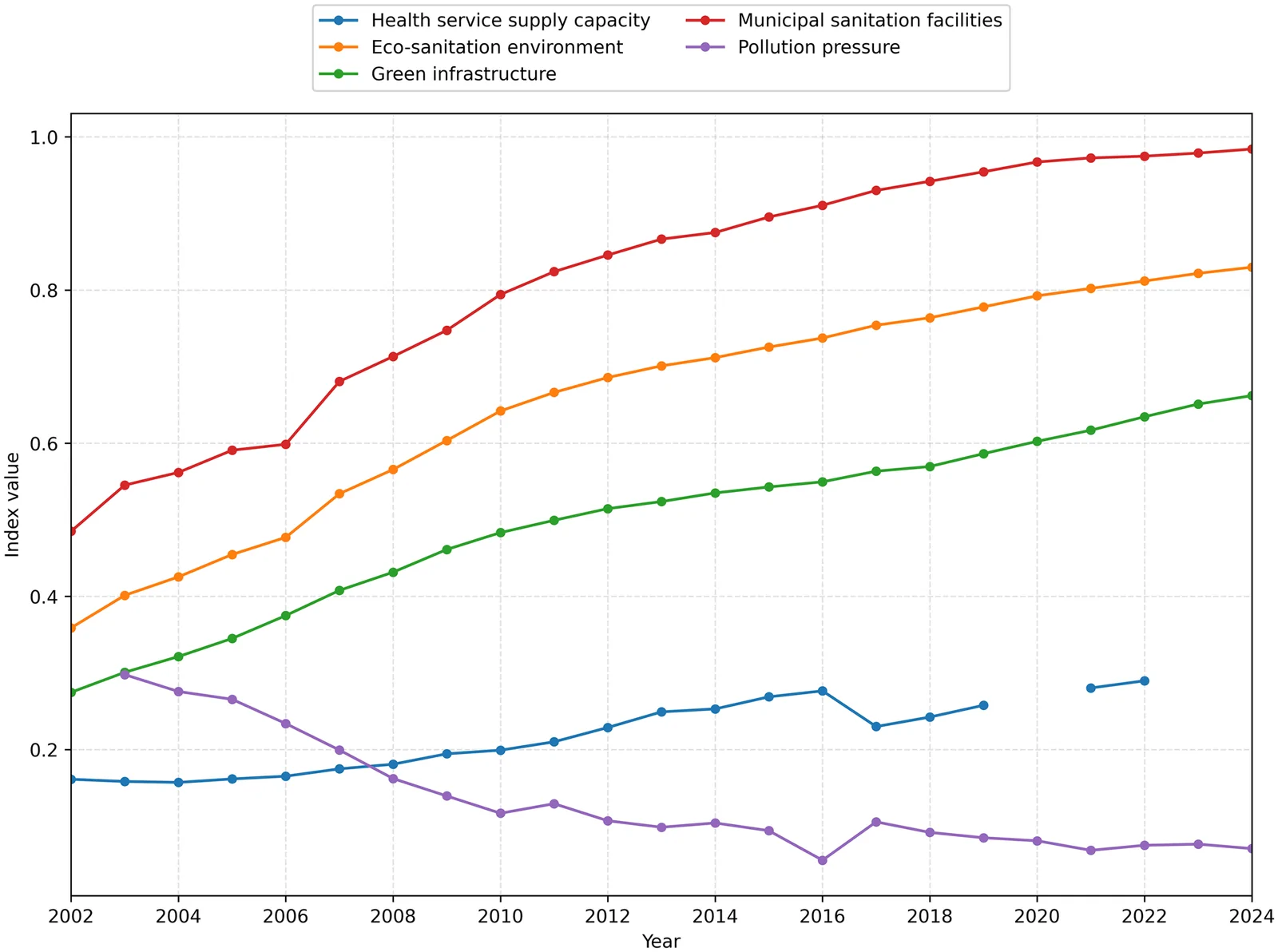
Temporal evolution of core indices

Figure 2 shows the annual evolution of the core indices during 2002–2024. The eco-sanitation environment, green infrastructure, and municipal sanitation facility indices generally increased, while the pollution pressure index generally declined, indicating evident improvement in urban eco-sanitation infrastructure and pollution governance. The health service supply capacity index also increased overall, but breaks appear due to missing health service statistical data in 2020, 2023, and 2024. Therefore, subsequent regional comparisons use 2022, the latest year with available health service supply capacity data.

**Fig. 3:**
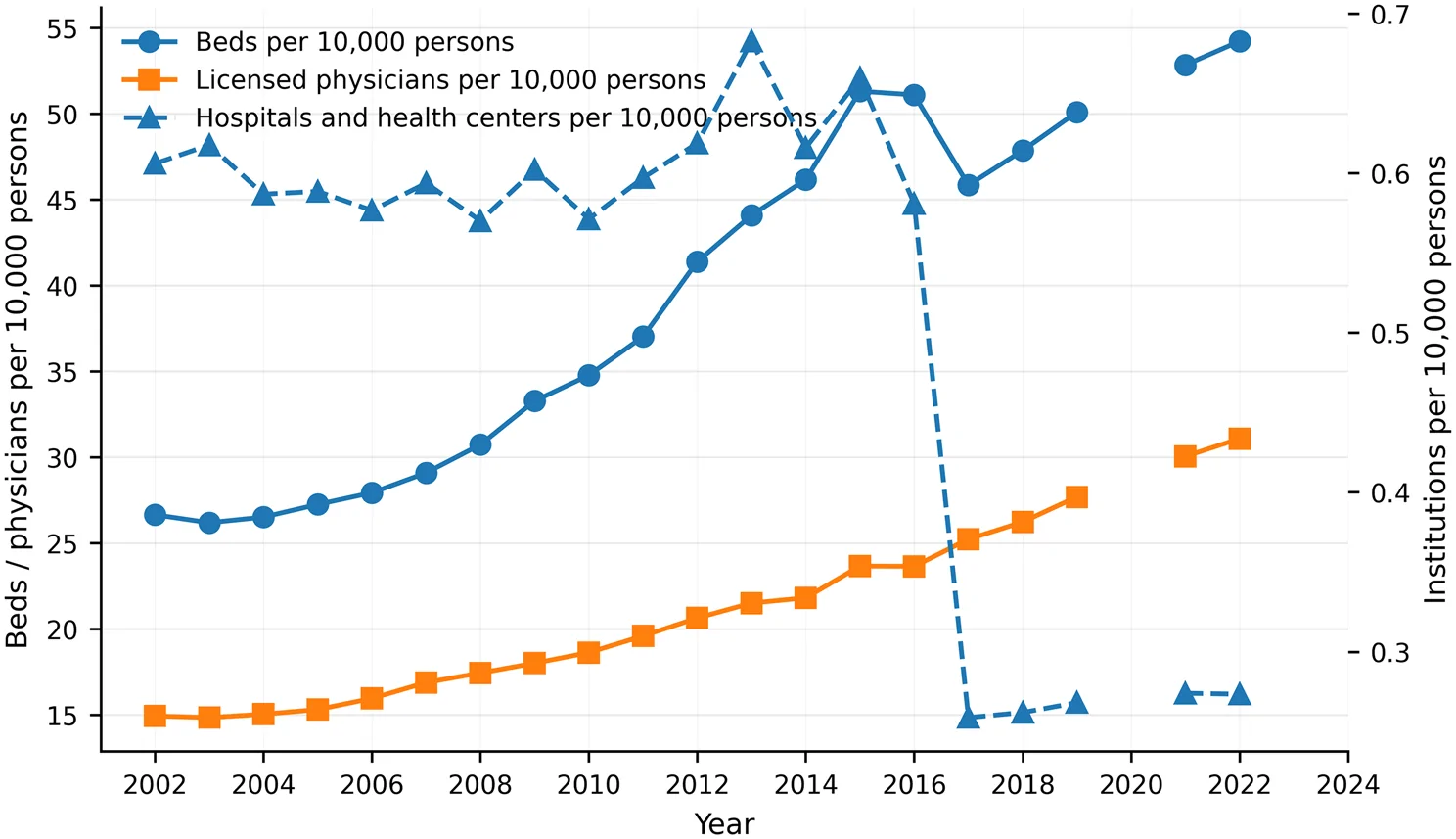
Temporal evolution of health service supply capacity components

In terms of the components of health service supply capacity, Figure 3 shows that hospital beds per 10,000 persons and licensed physicians per 10,000 persons increased continuously, indicating overall expansion of physical medical resources and health workforce resources. By contrast, the number of hospitals and health centers per 10,000 persons changed only modestly, suggesting that the improvement in health service supply capacity mainly came from the expansion of beds and physicians rather than from a simultaneous increase in the number of medical institutions.

**Fig. 4:**
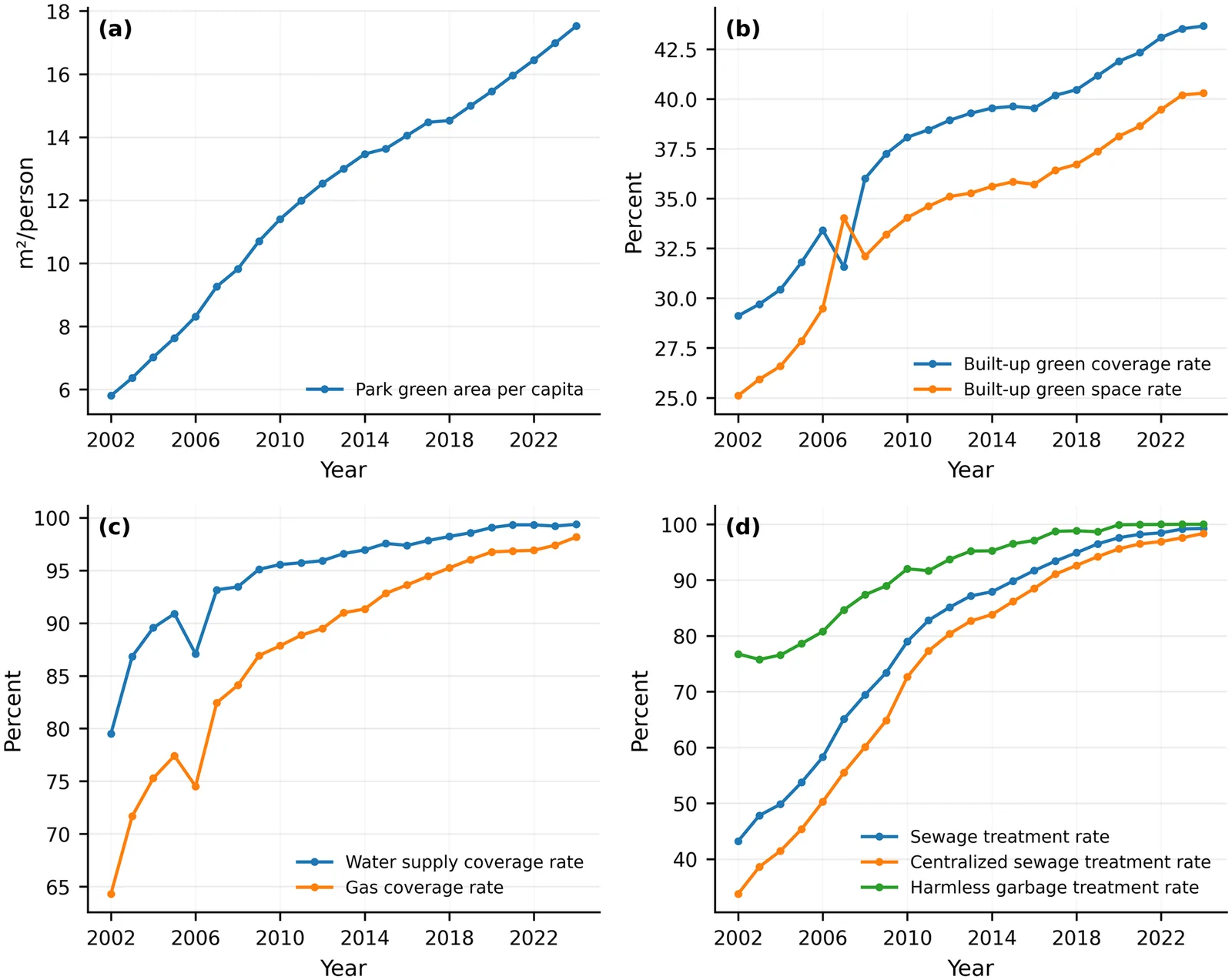
Temporal evolution of eco-sanitation environment components

Figure 4 further decomposes the component indicators of the eco-sanitation environment. Park green area per capita, built-up area green coverage rate, and built-up area green-space rate increased steadily, reflecting continuous improvement in green infrastructure. Water supply coverage and gas coverage remained at relatively high levels overall. Sewage treatment rate, centralized sewage treatment rate, and harmless domestic waste treatment rate increased more rapidly, indicating that municipal sanitation facilities were the main drivers of the increase in the eco-sanitation environment index.

**Fig. 5:**
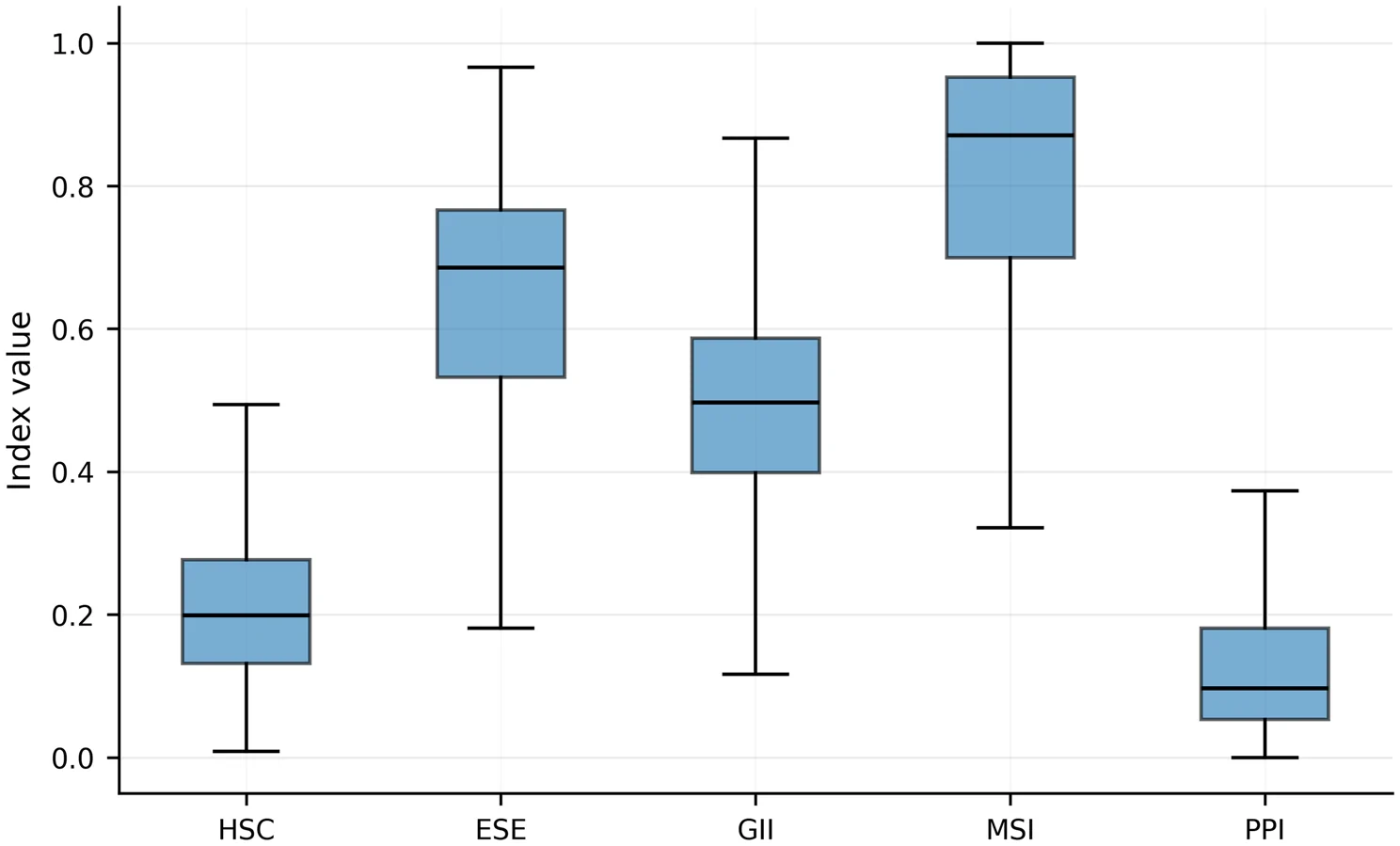
Distribution characteristics of main composite indices

Figure 5 presents the distribution of the main composite indices. The eco-sanitation environment and municipal sanitation facility indices have relatively high medians, and the latter is more concentrated. The health service supply capacity index and the green infrastructure index are more dispersed, indicating strong inter-city differences in health service resources and green infrastructure. The pollution pressure index is generally low but has a long right tail, suggesting that pollution pressure in a small number of cities was substantially higher than the sample average.

**Fig. 6:**
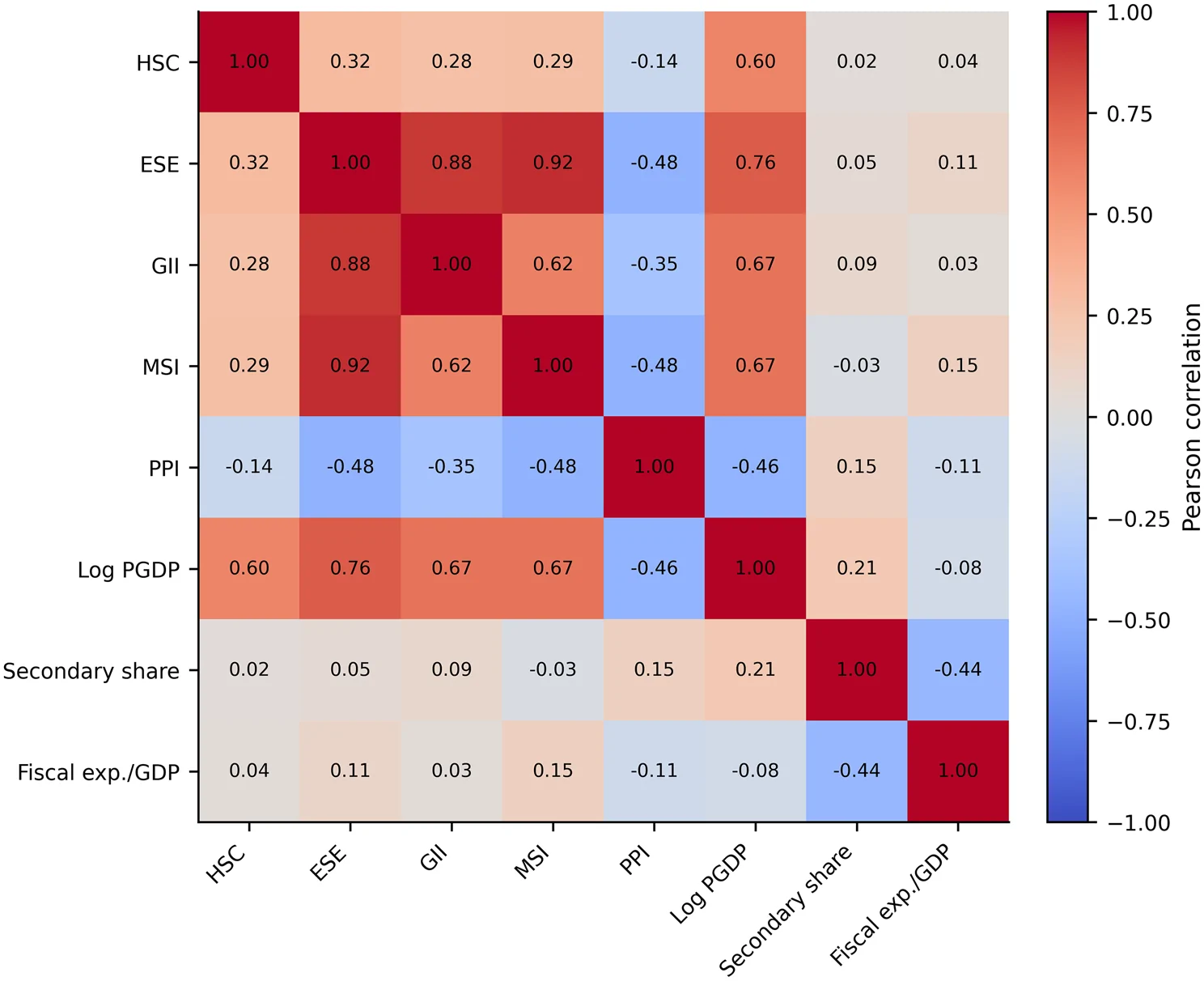
Correlation matrix of core variables

The correlation matrix shows that health service supply capacity is more strongly correlated with economic development, population size, and fiscal capacity, whereas its simple correlations with eco-sanitation variables are unstable (Fig. 6). This means that cross-sectional correlations alone cannot determine whether eco-sanitation improvement increases health service supply capacity; it is necessary to further control for unobserved city characteristics and common year shocks through city and year fixed effects.

**Fig. 7:**
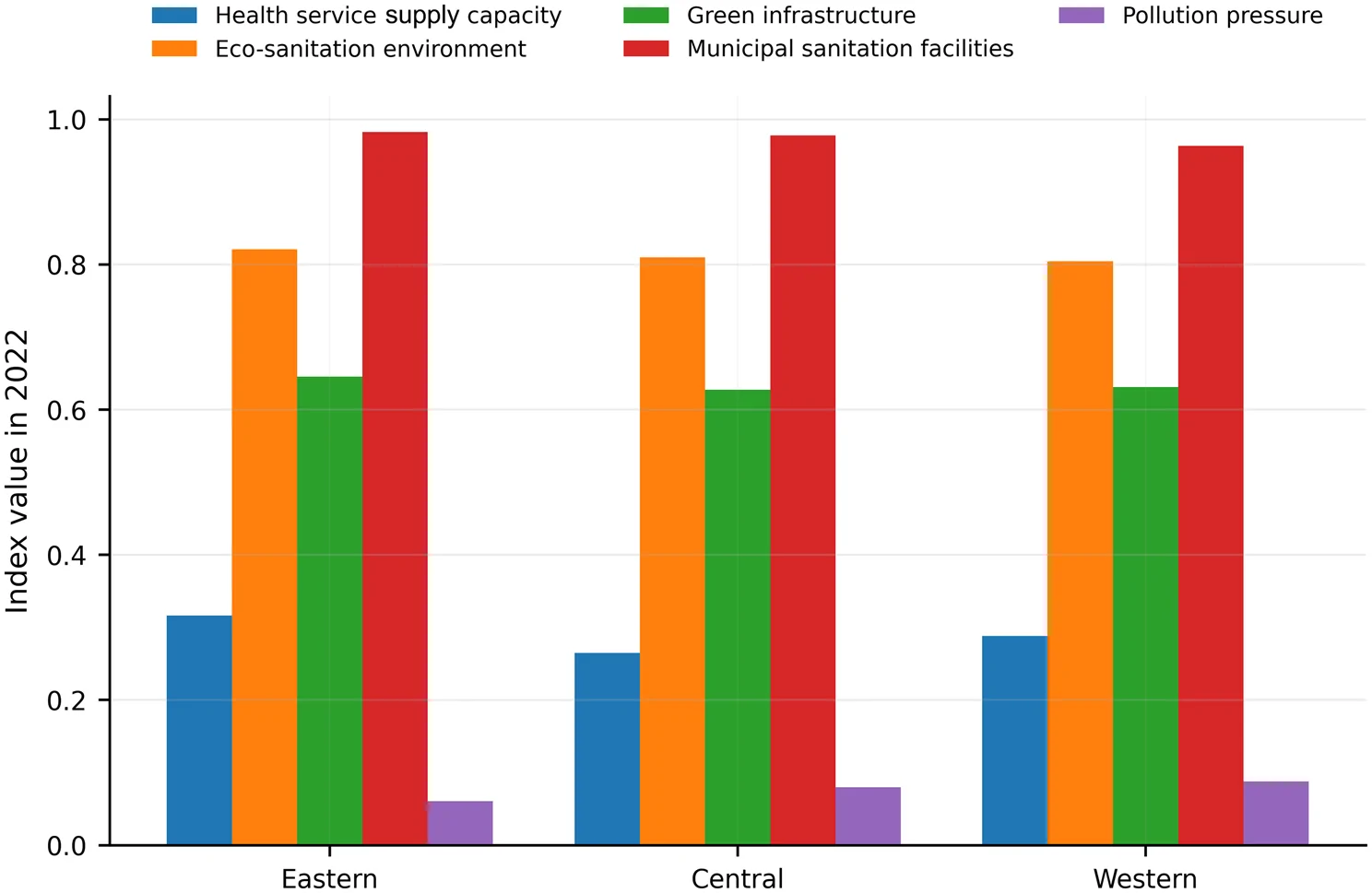
Regional comparison of core indices in 2022

Figure 7 compares the core indices across regions in 2022. Eastern cities had relatively higher health service supply capacity and higher levels of eco-sanitation environment and municipal sanitation facilities. Although the eco-sanitation environment differed less between central and western cities, their health service supply capacity remained lower than that of eastern cities. This result suggests that eco-sanitation improvement is not necessarily equivalent to synchronous improvement in health service supply capacity; the relationship between the two may be jointly shaped by economic development, population size, fiscal capacity, and the functional hierarchy of urban services.

**Fig. 8:**
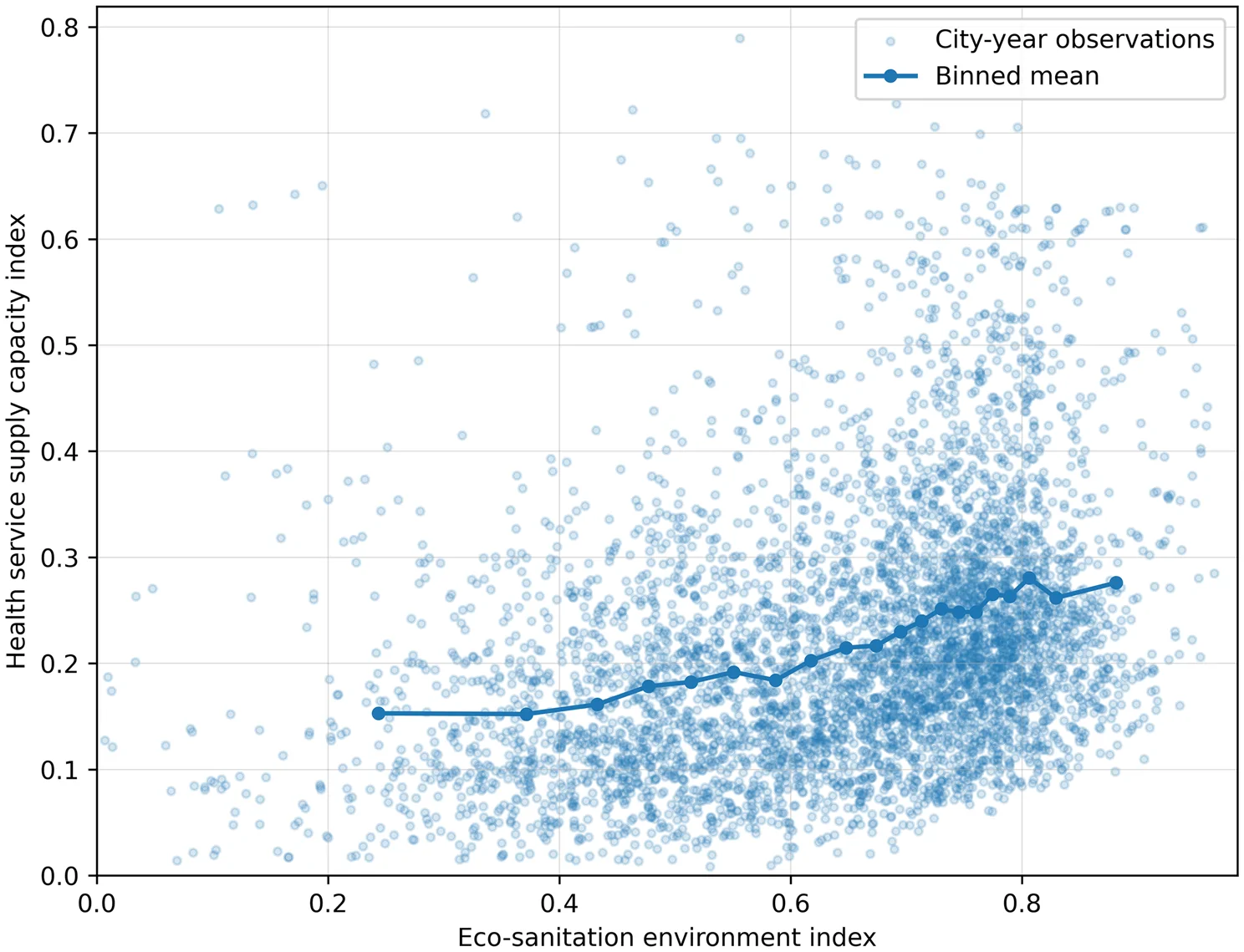
Binned mean relationship between the eco-sanitation environment and health service supply capacity

Figure 8 shows the scatter relationship and binned mean curve between the eco-sanitation environment and health service supply capacity. The two do not show a simple monotonic positive relationship. In the high range of the eco-sanitation environment index, the mean health service supply capacity levels off or even declines. This pattern is consistent with the specification of nonlinear and stage-dependent effects in Section “Methods” and provides preliminary evidence for the subsequent two-way fixed effects and interpretable machine learning analyses.

### Baseline relationship: does eco-sanitation improvement translate into health service supply capacity?

Table 6 reports the baseline two-way fixed effects estimates of the effect of lagged eco-sanitation environment on urban health service supply capacity. All models control for city fixed effects and year fixed effects, and standard errors are clustered at the city level. For ease of interpretation, the eco-sanitation environment index is scaled by 0.1 units.

**Table 6:** Baseline two-way fixed effects regression results

Variable	(1)	(2)	(3)	(4)
ESE (lagged, per 0.1 increase)	−0.0069*** (0.0020)	−0.0057*** (0.0020)	−0.0053*** (0.0020)	−0.0006 (0.0020)
Log per capita GDP	—	−0.0149 (0.0111)	−0.0123 (0.0130)	0.0015 (0.0163)
Secondary industry share	—	−0.0007** (0.0003)	−0.0004 (0.0005)	−0.0009 (0.0006)
Tertiary industry share	—	—	0.0003 (0.0006)	−0.0003 (0.0006)
Fiscal expenditure/GDP	—	−0.0936** (0.0425)	−0.1390*** (0.0464)	−0.0427 (0.0475)
Education expenditure/GDP	—	—	0.3025 (0.2523)	−0.1231 (0.2014)
Science expenditure/GDP	—	—	2.6187*** (0.8750)	0.7774 (0.5786)
Log population	—	—	−0.0266 (0.0382)	−0.0819** (0.0346)
Loan balance/GDP	—	—	0.0174** (0.0075)	0.0037 (0.0061)
Fixed asset investment/GDP	—	—	—	0.0153 (0.0109)
City FE	Yes	Yes	Yes	Yes
Year FE	Yes	Yes	Yes	Yes
Observations	5070	5070	5069	3919
Cities	288	288	288	285
Adjusted R^2^	0.011	0.025	0.042	0.011
Note	Robust standard errors clustered at city level are shown in parentheses. *** *p* < 0.01, ** *p* < 0.05, * *p* < 0.10.	—	—	—

**Fig. 9:**
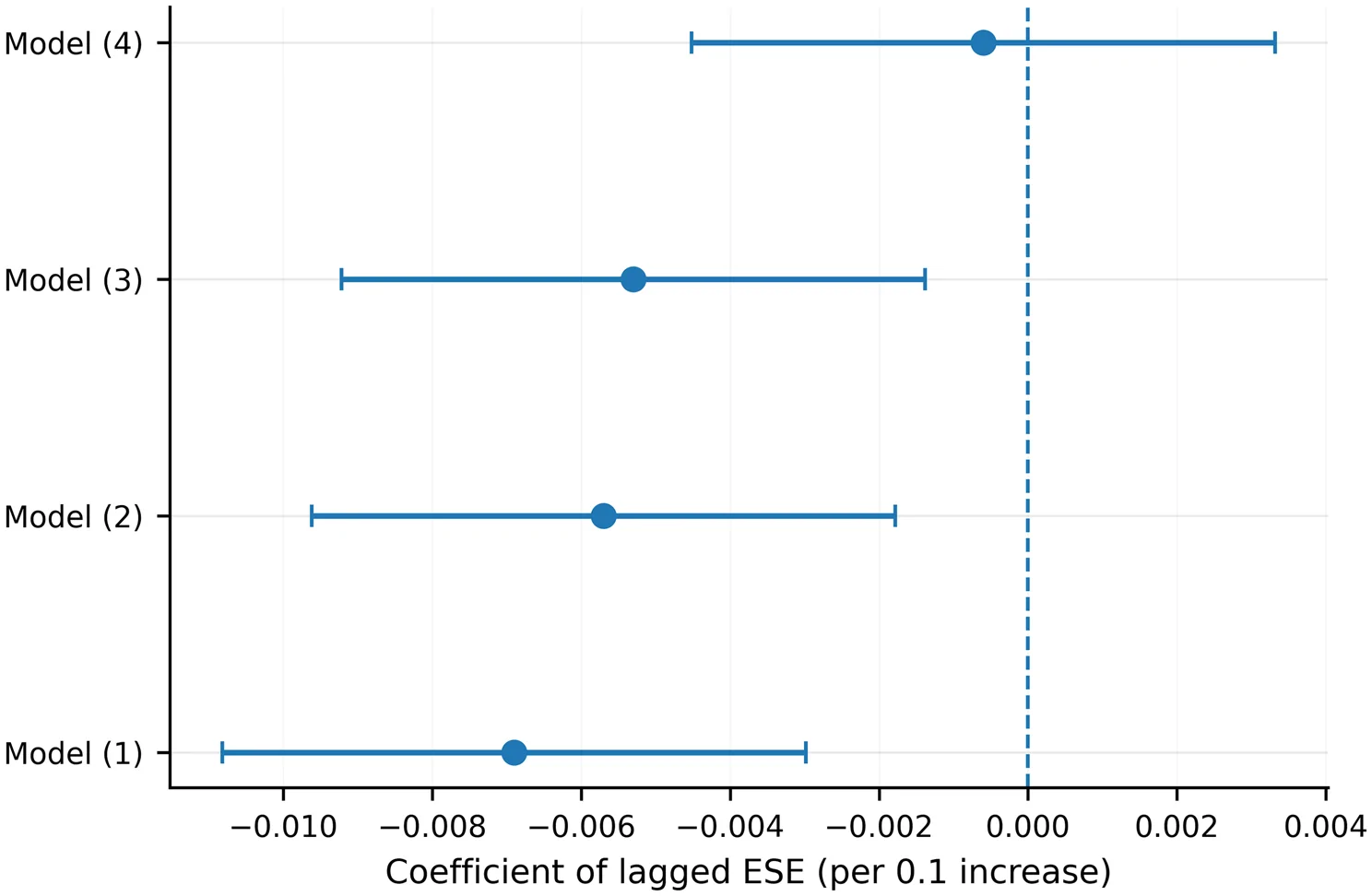
Coefficient estimates of the lagged eco-sanitation environment index in baseline models

The results show that in model (1), which controls only for city and year fixed effects, the coefficient of the lagged eco-sanitation environment index is −0.0069 and significant at the 1% level. After adding baseline controls such as economic development, industrial structure, and fiscal capacity, the coefficient in model (2) is −0.0057 and remains significant at the 1% level. After further adding extended controls such as the tertiary industry share, education expenditure, science and technology expenditure, population size, and financial development, the coefficient in model (3) is −0.0053 and remains significant. When fixed-asset investment as a share of GDP is included, the sample size falls to 3,919 observations and the coefficient becomes insignificant. Figure 9 visualizes this change in coefficients.

The negative coefficient should not be interpreted as showing that a better ecological environment causes poorer health-service capacity. First, ESE is a composite measure of green-space and municipal sanitation infrastructure coverage, rather than a complete measure of environmental quality or population health. Second, the coefficient is a conditional within-city association: in model (2), a 0.1-unit increase in lagged ESE is associated with a 0.0057 decrease in HSC, equivalent to approximately 2.6% of the sample mean HSC (0.2201), after controlling for observed covariates and city and year fixed effects. Third, the estimate attenuates as controls are expanded and becomes statistically insignificant in model (4), indicating sensitivity to model specification and sample composition. The result is therefore interpreted as evidence of an asynchronous conversion or resource-allocation mismatch—eco-sanitation infrastructure may improve before beds, physicians, and service outlets adjust—rather than as evidence that environmental improvement damages health services or residents’ health.

To address the decline in sample size from 5,070 to 3,919 observations in model (4), this study conducted additional tests of missing-sample characteristics. Compared with excluded observations, the sample retained in model (4) has a lower mean health service supply capacity (0.206 vs. 0.268), lower lagged eco-sanitation environment (0.571 vs. 0.778), lower log per capita GDP (10.079 vs. 11.004), and lower loan balance/GDP (0.821 vs. 1.277), but a higher secondary industry share (48.482 vs. 40.374). Most of these differences are statistically significant, indicating that the missingness in model (4) is not completely random. Therefore, the coefficient becoming insignificant in model (4) should not be interpreted as a direct rejection of the baseline result, but as a supplementary test on a non-randomly reduced sample after adding the fixed-asset investment variable.

These results indicate that after controlling for unobserved time-invariant city characteristics and common national year shocks, eco-sanitation improvement was not stably converted into stronger health service supply capacity. This finding should not be interpreted as evidence that eco-sanitation improvement weakens health service capacity. Rather, it more likely reflects two forms of conversion mismatch: first, a time lag between eco-sanitation infrastructure construction and healthcare resource allocation; and second, marginally diminishing room for eco-sanitation improvement in some cities with already high health service supply capacity. Thus, RQ1 does not show a stable positive conversion relationship.

### Dimension-specific differences: different roles of green infrastructure, municipal sanitation facilities, and pollution pressure

**Fig. 10:**
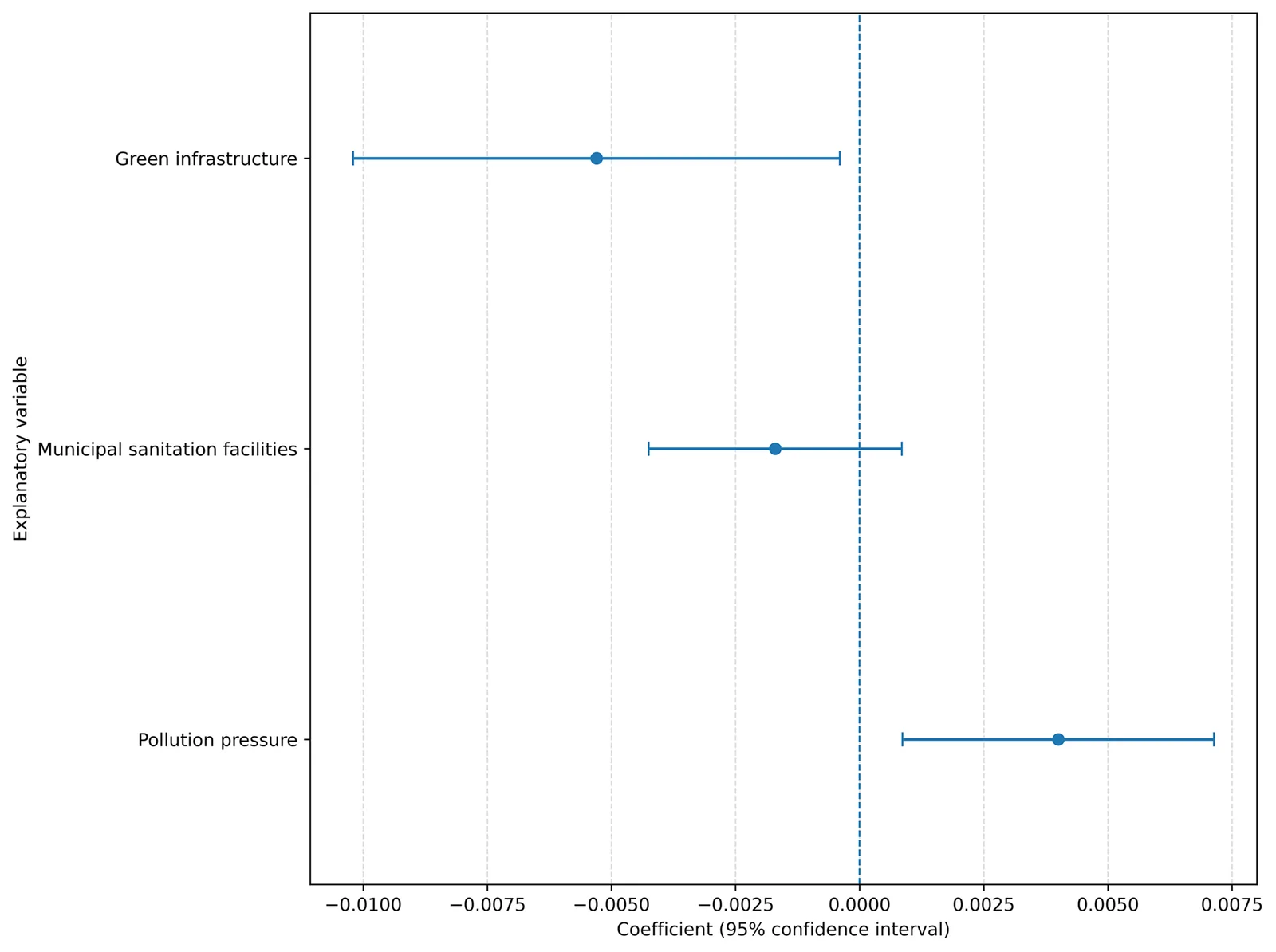
Dimension-specific coefficients of green infrastructure, municipal sanitation facilities, and pollution pressure

To identify the roles of different dimensions within the eco-sanitation environment, this study decomposes it into green infrastructure, municipal sanitation facilities, and pollution pressure. Figure 10 shows that when the three variables enter the model simultaneously, the coefficient of green infrastructure is significantly negative, the coefficient of municipal sanitation facilities is no longer significant, and the coefficient of pollution pressure is significantly positive.

The dimension-specific estimates show that ESE is not a simple linear composite of uniformly beneficial components. In the single-dimension model, a 0.1 increase in lagged PPI is associated with a 0.0034 increase in HSC (SE = 0.0015, *p* < 0.05); in the joint model the coefficient is 0.0040 (SE = 0.0016, *p* < 0.05). These positive coefficients must not be interpreted as evidence that pollution improves health, healthcare quality, or population well-being. They are associational estimates and are more plausibly consistent with a demand-induced or compensatory resource-allocation response: highly industrialized and populous cities may face greater latent healthcare demand while also possessing stronger fiscal and organizational capacity to allocate beds, physicians, and institutions. The result concerns observed supply responses under pollution pressure, not a beneficial causal effect of pollution itself.

To examine the risk of multicollinearity when the three dimension-specific indicators enter the model simultaneously, this study calculated variance inflation factors (VIFs). The VIFs of the green infrastructure, municipal sanitation facility, and pollution pressure indices are 1.96, 2.39, and 1.58, respectively. After including the main control variables, the VIFs of all core eco-sanitation variables remain below 3, and the maximum VIF among all explanatory variables is 5.05, below the commonly used empirical threshold of 10. Thus, the dimension-specific results are unlikely to be mainly driven by severe multicollinearity.

### Conversion channel test: does urban construction investment form an effective transmission pathway?

**Fig. 11:**
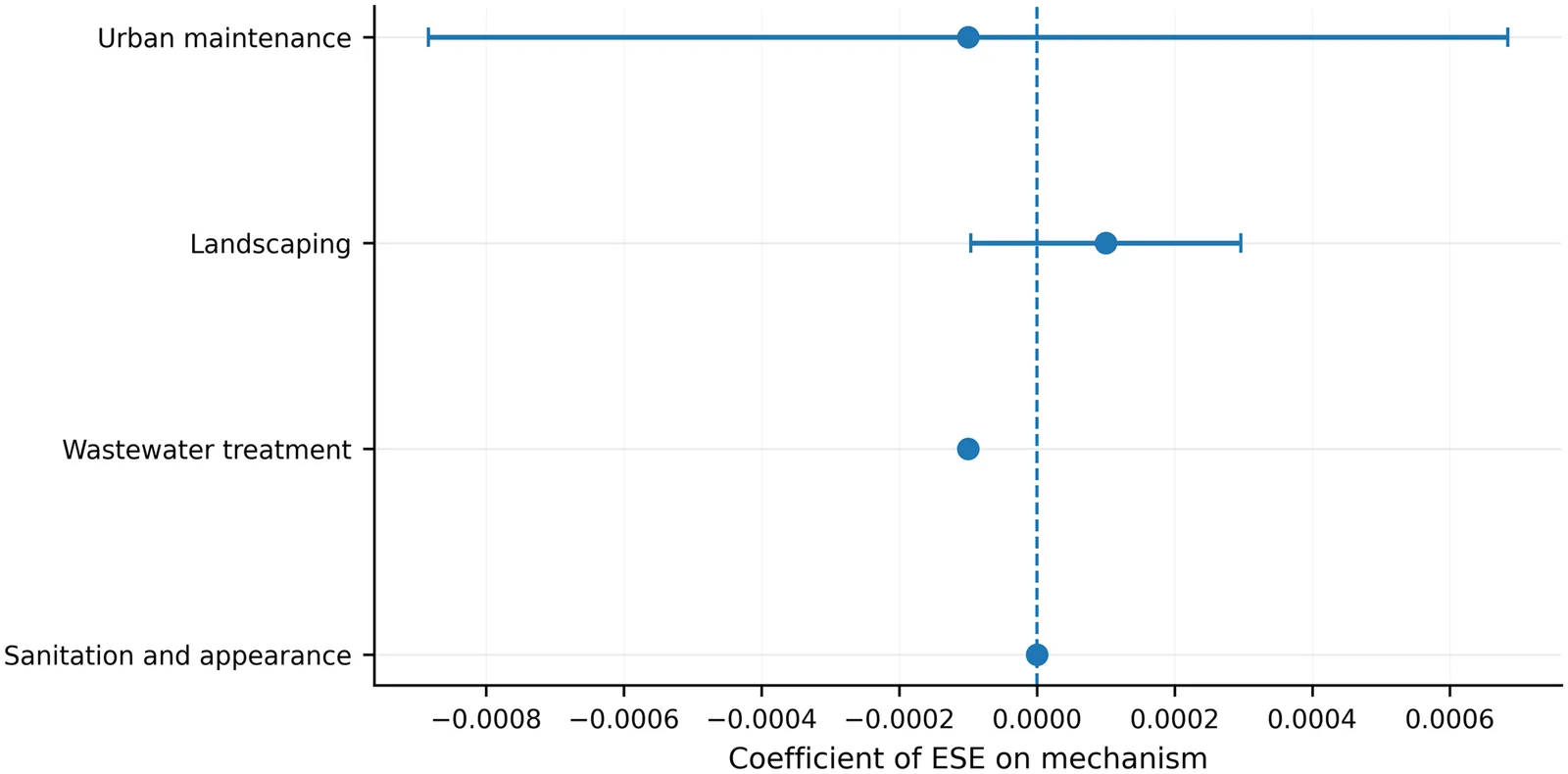
First-stage effects of the eco-sanitation environment on mechanism variables

**Fig. 12:**
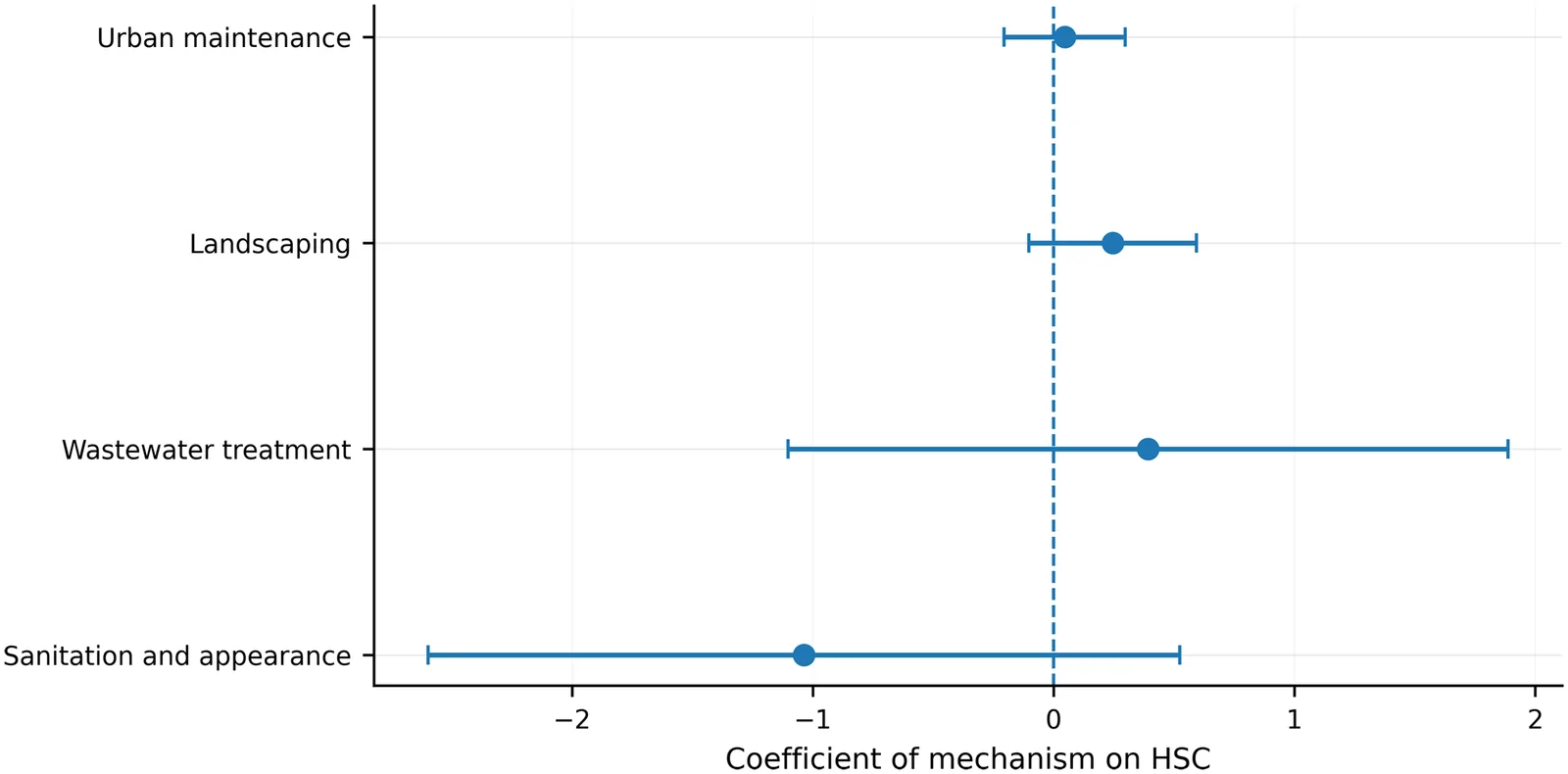
Direct effects of mechanism variables on health service supply capacity

Section “Methods” narrowed the mechanism pathway to five project-specific investment channels directly related to the components of the eco-sanitation environment and urban public infrastructure provision: urban maintenance and construction expenditure as a share of GDP, landscaping investment as a share of GDP, wastewater treatment investment as a share of GDP, sanitation and environmental appearance investment as a share of GDP, and garbage-treatment investment as a share of GDP. Local fiscal expenditure as a share of GDP is retained separately as a broader fiscal-capacity indicator in the original one-year mechanism analysis. Figures 11 and 12 visualize the four core project-specific variables for readability. The eco-sanitation environment has a weak positive association with landscaping investment, a significant negative association with wastewater treatment investment, and insignificant effects on urban maintenance and construction expenditure and sanitation and environmental appearance investment. The longer-lag extension reported in Table 7 is limited to the five project-specific investment variables.

Figure 12 presents the original one-year mechanism specifications. The contemporaneous investment and expenditure variables do not have stable direct associations with HSC, and the ESE coefficient remains mostly negative after their inclusion. Because healthcare resource planning may require more than one year, the five project-specific investment channels were extended to two- and three-year ESE lags and to one- and two-year lagged investment variables. The original one-year estimates are therefore treated as the starting specification rather than as the sole basis for rejecting a delayed pathway.

Table 7 reports the extended lag tests for the five project-specific investment variables; local fiscal expenditure/GDP is not included because it is treated as the broader fiscal-capacity indicator in the original one-year specification. With two-year ESE lags, ESE is negatively associated only with wastewater-treatment investment (−0.0010, SE = 0.0005, *p* = 0.0499), while all mechanism-to-HSC coefficients are insignificant. With three-year ESE lags, the wastewater association is only weakly significant (−0.0007, SE = 0.0004, *p* = 0.099), and again no mechanism variable predicts HSC at conventional levels. When mechanism variables are lagged, only one-year lagged landscaping investment is weakly positive (0.2693, SE = 0.1558, *p* = 0.085), but this association disappears at the two-year lag. Across the remaining investment measures, one- and two-year lagged effects are insignificant. Thus, extending the lag structure does not reveal a stable multi-year transmission chain.

**Table 7:** Extended project-specific investment mechanism tests with longer lags

Panel / lag	Mechanism variable	ESE → mechanism	Mechanism → HSC	ESE after mechanism	N
A: ESE lag 2	Urban maintenance expenditure/GDP	−0.0030 (0.0032)	−0.0303 (0.1251)	−0.0047 (0.0204)	3617
A: ESE lag 2	Landscaping investment/GDP	−0.0006 (0.0008)	0.1783 (0.1716)	−0.0601*** (0.0225)	4328
A: ESE lag 2	Wastewater treatment investment/GDP	−0.0010** (0.0005)	0.2120 (0.7264)	−0.0603*** (0.0202)	3179
A: ESE lag 2	Sanitation and appearance investment/GDP	−0.0002 (0.0002)	−1.1856 (0.7355)	−0.0551** (0.0228)	3866
A: ESE lag 2	Garbage treatment investment/GDP	−0.0002 (0.0002)	−1.3119 (1.4585)	−0.0522*** (0.0195)	2462
A: ESE lag 3	Urban maintenance expenditure/GDP	−0.0037 (0.0029)	−0.0528 (0.1234)	−0.0076 (0.0192)	3338
A: ESE lag 3	Landscaping investment/GDP	−0.0033 (0.0023)	0.0794 (0.1642)	−0.0718*** (0.0218)	4061
A: ESE lag 3	Wastewater treatment investment/GDP	−0.0007* (0.0004)	0.6063 (0.7513)	−0.0748*** (0.0200)	2996
A: ESE lag 3	Sanitation and appearance investment/GDP	−0.0001 (0.0002)	−0.8809 (0.7619)	−0.0635*** (0.0224)	3612
A: ESE lag 3	Garbage treatment investment/GDP	0.0000 (0.0002)	−0.5508 (1.5329)	−0.0636*** (0.0198)	2287
B: M lag 1	Urban maintenance expenditure/GDP	—	0.0793 (0.1430)	−0.0077 (0.0205)	3897
B: M lag 1	Landscaping investment/GDP	—	0.2693* (0.1558)	−0.0602*** (0.0219)	4568
B: M lag 1	Wastewater treatment investment/GDP	—	0.6514 (0.7922)	−0.0559** (0.0220)	3320
B: M lag 1	Sanitation and appearance investment/GDP	—	−0.7574 (0.9320)	−0.0669*** (0.0223)	4115
B: M lag 1	Garbage treatment investment/GDP	—	0.9353 (1.5731)	−0.0685*** (0.0210)	2617
B: M lag 2	Urban maintenance expenditure/GDP	—	−0.0431 (0.1314)	−0.0238 (0.0216)	3893
B: M lag 2	Landscaping investment/GDP	—	0.0273 (0.1817)	−0.0734*** (0.0236)	4280
B: M lag 2	Wastewater treatment investment/GDP	—	0.8575 (0.8986)	−0.0600** (0.0265)	3024
B: M lag 2	Sanitation and appearance investment/GDP	—	−1.0789 (0.9145)	−0.0728*** (0.0254)	3824
B: M lag 2	Garbage treatment investment/GDP	—	0.7984 (1.1484)	−0.0740*** (0.0203)	2348

Note: City and year fixed effects and baseline controls are included; standard errors are clustered by city. Panels A and B cover the five project-specific investment variables listed in the table. Local fiscal expenditure/GDP is retained in the original one-year mechanism specification as a broader fiscal-capacity indicator and is not included in this longer-lag extension. Panel A varies the lag of ESE. Panel B varies the lag of the mechanism variable and retains ESE(t-1). * *p* < 0.10, ** *p* < 0.05, *** *p* < 0.01

### Moderating effects of energy and carbon emissions

**Fig. 13:**
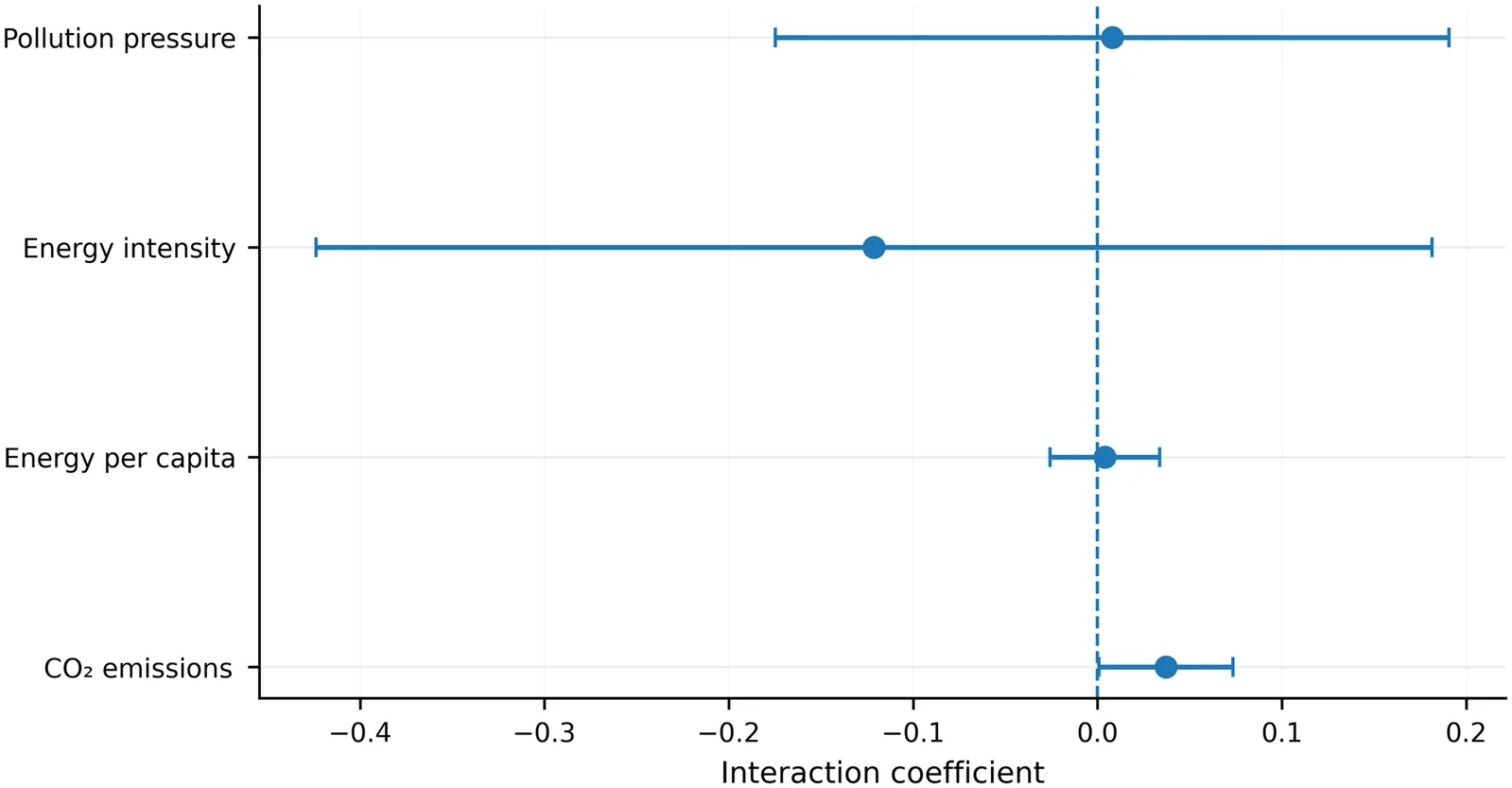
Moderating effects of pollution pressure, energy intensity, and carbon emissions

Figure 13 reports the main moderation specifications. Pollution pressure, energy intensity, and per capita energy consumption interactions are insignificant. The carbon interaction in the original jointly available covariate sample (*N* = 2,396; 249 cities) is positive and significant at the 5% level. Because CEADs ends in 2019, this estimate does not cover pandemic or post-pandemic health-resource shifts and is not interpreted as evidence for 2020–2024.

The positive carbon interaction is more plausibly interpreted as the resource-allocation capacity and public-service demand associated with industrial and high-emission cities, rather than as a beneficial effect of emissions. To separate sample composition from the carbon interaction, the model was re-estimated on the exact CEADs moderation sample. Table 8 shows that the baseline ESE coefficient is −0.0582 (SE = 0.0190, *p* = 0.002) and the ESE×CO₂ interaction is 0.0011 (SE = 0.0004, *p* = 0.018; *N* = 3,147; 255 cities). A baseline model for the same CEADs-matched cities over 2002–2019 gives −0.0388 (SE = 0.0226, *p* = 0.087; *N* = 4,045). These comparisons support the presence of a positive pre-2020 interaction but also show that magnitude and precision depend on sample construction. The result is therefore explicitly limited to pre-pandemic evidence.

**Table 8:** Ceads exact-sample and temporal-comparability tests

Comparable-sample model	Key coefficient	Coefficient (SE)	N	Cities	Interpretive scope
Baseline on exact moderation sample	ESE(*t*-1)	−0.0582*** (0.0190)	3147	255	2002–2019; pre-pandemic
Moderation on exact CEADs sample	ESE × CO₂	0.0011** (0.0004)	3147	255	2002–2019; pre-pandemic
Baseline, CEADs-matched cities	ESE(*t*-1)	−0.0388* (0.0226)	4045	255	2002–2019; pre-pandemic

Note: All models include city and year fixed effects, baseline controls, and city-clustered standard errors. The CEADs series does not observe pandemic-related or post-2019 health-resource changes

### Heterogeneity analysis

**Fig. 14:**
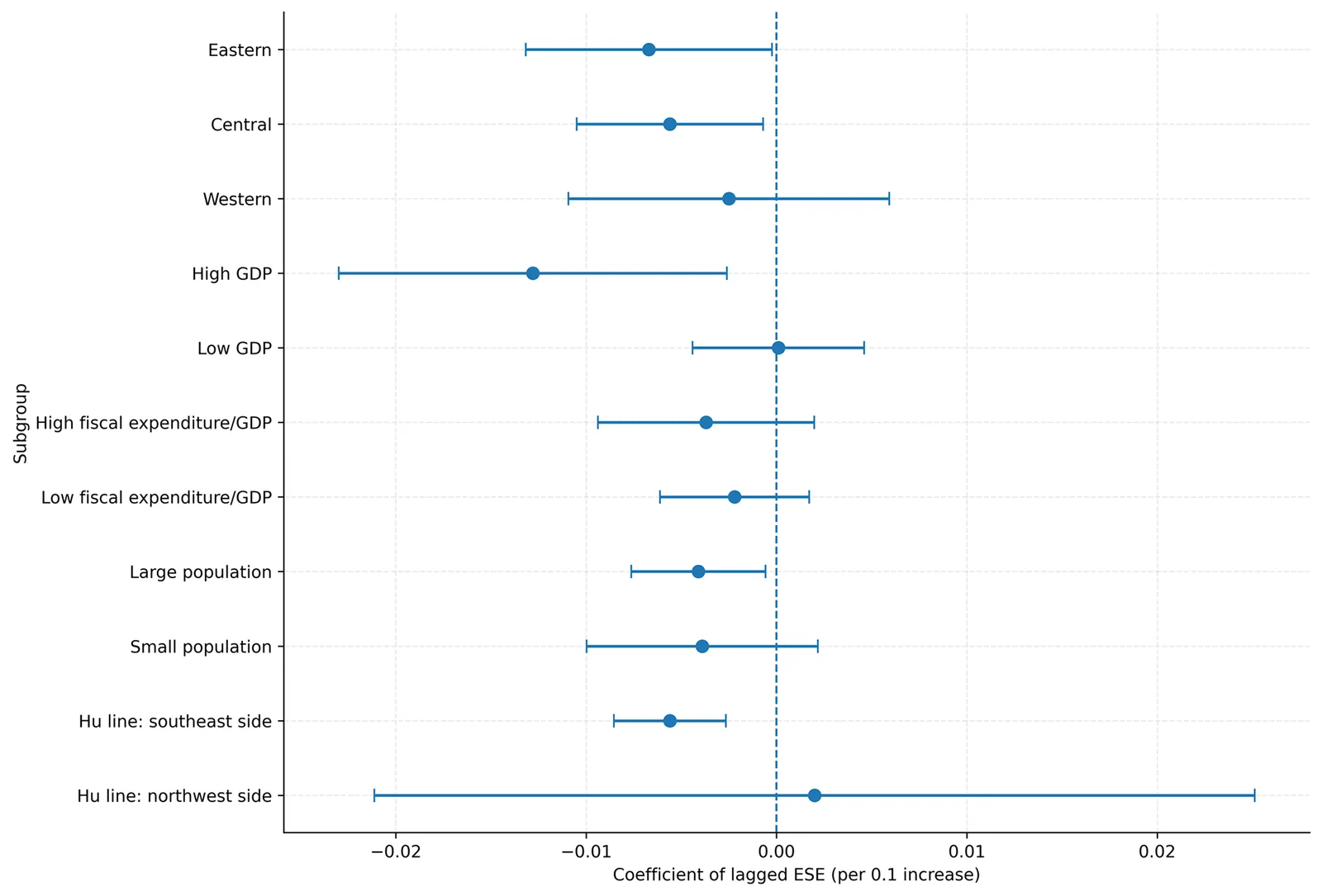
Heterogeneous coefficients of the eco-sanitation environment across city types

Figure 14 reports the primary city-and-year fixed-effects heterogeneity estimates. Regional coefficients are significantly negative in eastern and central cities but insignificant in western cities; the coefficient is significant in high-GDP and large-population groups but not in their comparison groups. In the primary Hu-line grouping, the southeastern coefficient is negative and significant, whereas the northwestern coefficient is insignificant.

The regional, economic-development, and population-size patterns indicate that the ESE-HSC relationship varies with urban development and resource-allocation conditions. However, the Hu Huanyong line should not be interpreted as an independent policy-free geographic effect. After province-year fixed effects are added, the southeastern coefficient attenuates from −0.0561 (SE = 0.0147, *p* < 0.001) to −0.0270 (SE = 0.0166, *p* = 0.105), while the northwestern coefficient remains insignificant (0.0671, SE = 0.1478, *p* = 0.653). Thus, the initial southeastern significance is partly absorbed by time-varying provincial policies and shocks. The stricter results support heterogeneity in urban context but do not establish an independent Hu-line effect. The corresponding subgroup estimates are reported in Table 9.

**Table 9:** Hu Huanyong line subgroup models with province-year fixed effects

Hu-line group	Fixed-effects specification	ESE(t-1) coefficient (SE)	N	Cities
Southeast side	City + year FE	−0.0561*** (0.0147)	4597	259
Southeast side	City + province-year FE	−0.0270 (0.0166)	4597	259
Northwest side	City + year FE	0.0197 (0.1181)	473	29
Northwest side	City + province-year FE	0.0671 (0.1478)	473	29

Note: Coefficients are reported for a one-unit change in ESE. All models include baseline controls and city-clustered standard errors. Province-year fixed effects absorb time-varying provincial and national shocks; common year fixed effects are not separately included in that specification

### Robustness checks

**Fig. 15:**
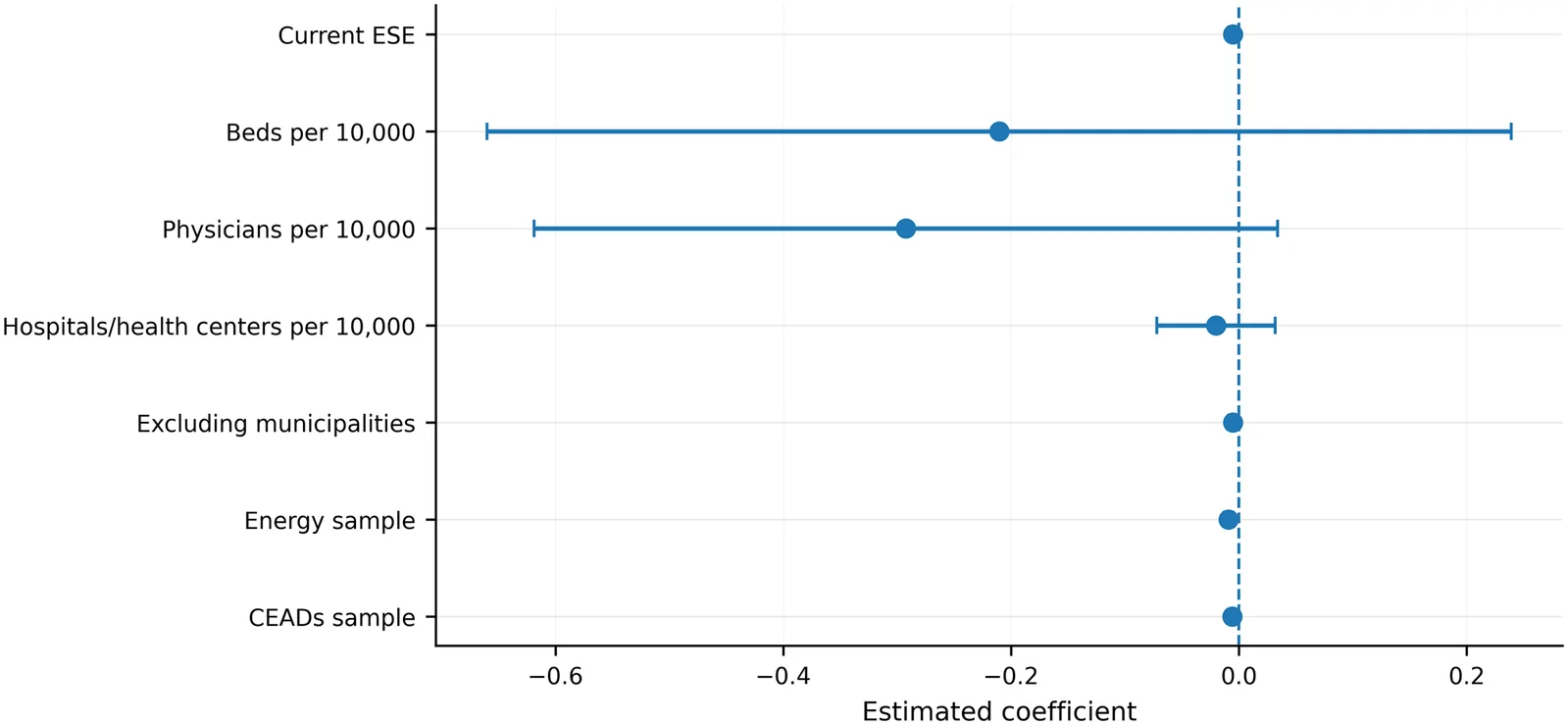
Core coefficient estimates in robustness checks

Figure 15 shows the main robustness-check results. When the lagged eco-sanitation environment is replaced with contemporaneous eco-sanitation environment, the coefficient remains significantly negative. When the dependent variable is replaced with the three component indicators of health service supply capacity, the eco-sanitation environment has a weakly significant negative effect on the number of licensed physicians per 10,000 persons, but insignificant effects on beds and institutional density. After excluding municipalities, the core coefficient remains significant. In the energy-extended and CEADs extended samples, the eco-sanitation environment coefficient is also significantly negative.

The robustness checks indicate that the baseline results are not driven by a specific sample, municipalities, or the lag specification. However, results using alternative dependent variables also suggest that the association between the eco-sanitation environment and the composite health service supply capacity index mainly arises from changes in health workforce and the composite index rather than from a single indicator such as beds or number of institutions. This further indicates that urban health service supply capacity is a multidimensional supply-side capacity and cannot be explained by a single health resource indicator. It should be emphasized that because this study uses city panel fixed effects models, the results mainly represent directional evidence under within-city changes rather than strict natural-experiment causal effects.

Further robustness checks show that when the entropy-weighted eco-sanitation environment index is replaced with an equal-weighted index, the core coefficient is −0.0050 (SE = 0.0018) and significant at the 1% level. When it is replaced with a PCA index, the core coefficient is −0.0035 (SE = 0.0017) and significant at the 5% level, indicating that the results do not depend on a single weighting method. With two-way clustered standard errors by city and year, the core coefficient remains −0.0053 (SE = 0.0026) and significant at the 5% level, indicating that the standard error specification does not change the main conclusion. For the placebo test, the coefficient of future one-year ESE is −0.0042 (SE = 0.0021) and is statistically significant at the 5% level. After including one-year, two-year, and three-year leads of ESE, the Wald statistic for the joint test of multi-period leads is 7.53, with a *p*-value of 0.057. The joint test does not pass the 5% significance level, but is close to the 10% level. This result suggests that the risk of pre-trends is weaker than indicated by the single lead test, but unobserved dynamic factors still cannot be completely excluded. Therefore, the baseline estimates are interpreted as conditional within-city associations and directional evidence rather than strict causal effects.

### Interpretable machine learning results

**Fig. 16:**
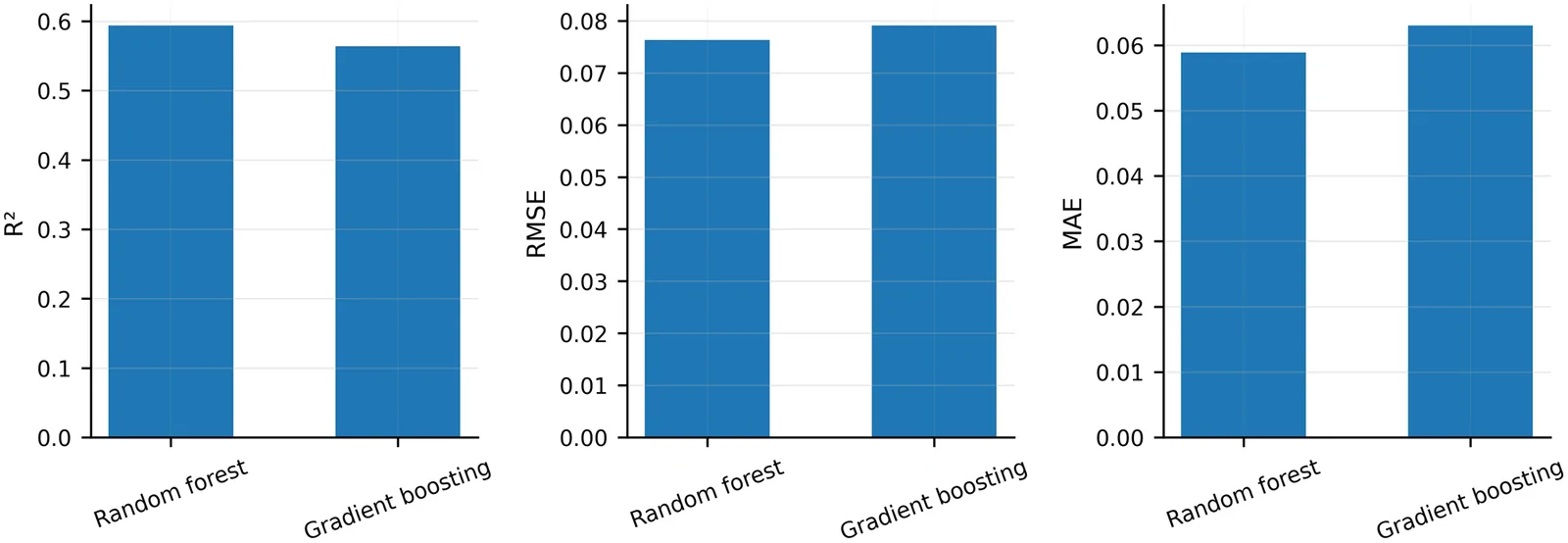
Comparison of predictive performance of machine learning models

Table 10 reports the predictive performance of the machine learning models. To avoid temporal leakage, observations from 2018 and earlier are used as the training set, and observations from 2019 onward are used as the test set. The random forest model achieves an R^2^ of 0.5936, an RMSE of 0.0763, and an MAE of 0.0589 on the test set, outperforming the gradient boosting model. Model hyperparameters are selected using the time-blocked cross-validation procedure described in Section “Methods” and then fixed to ensure reproducibility of variable importance and SHAP explanations. Figure 16 further shows that the random forest model has better overall performance in explaining nonlinear differences in urban health service supply capacity.

**Table 10:** Predictive performance of machine learning models

Model	R^2^	RMSE	MAE	Train N	Test N
Random forest	0.5936	0.0763	0.0589	3665	793
Gradient boosting	0.5637	0.0791	0.0630	3665	793

**Fig. 17:**
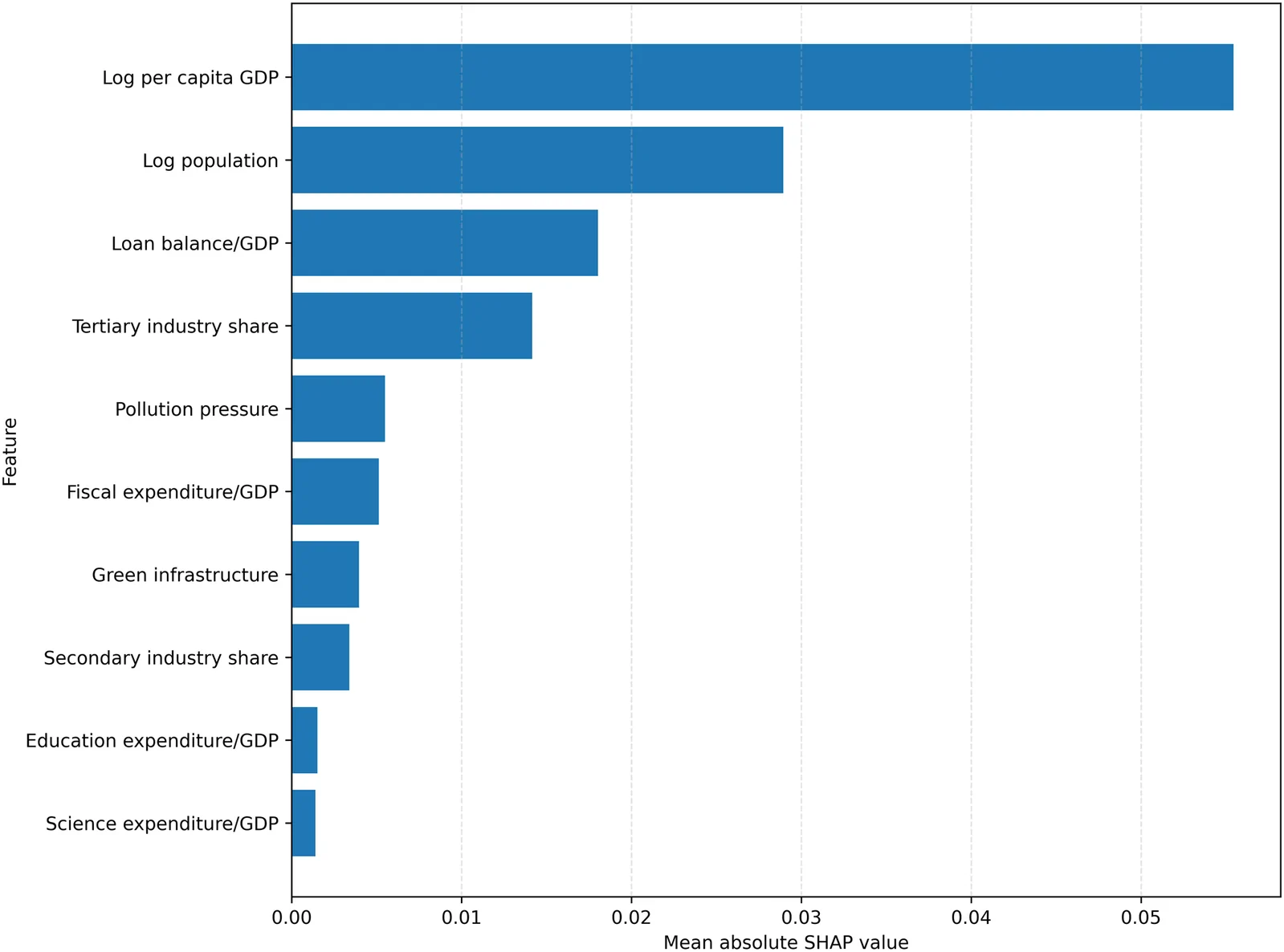
SHAP variable importance ranking

Figure 17 reports SHAP-based variable importance for the random forest model. The results show that per capita GDP, population size, the tertiary industry share, and loan balance as a share of GDP are the most important variables explaining differences in urban health service supply capacity. The importance of eco-sanitation-related variables is lower than that of economic and population variables, but pollution pressure and green infrastructure still rank among the explanatory features, indicating that eco-sanitation variables contribute to explaining health service supply capacity, although their strength is weaker than that of economic development, population size, and industrial-structure factors.

**Fig. 18:**
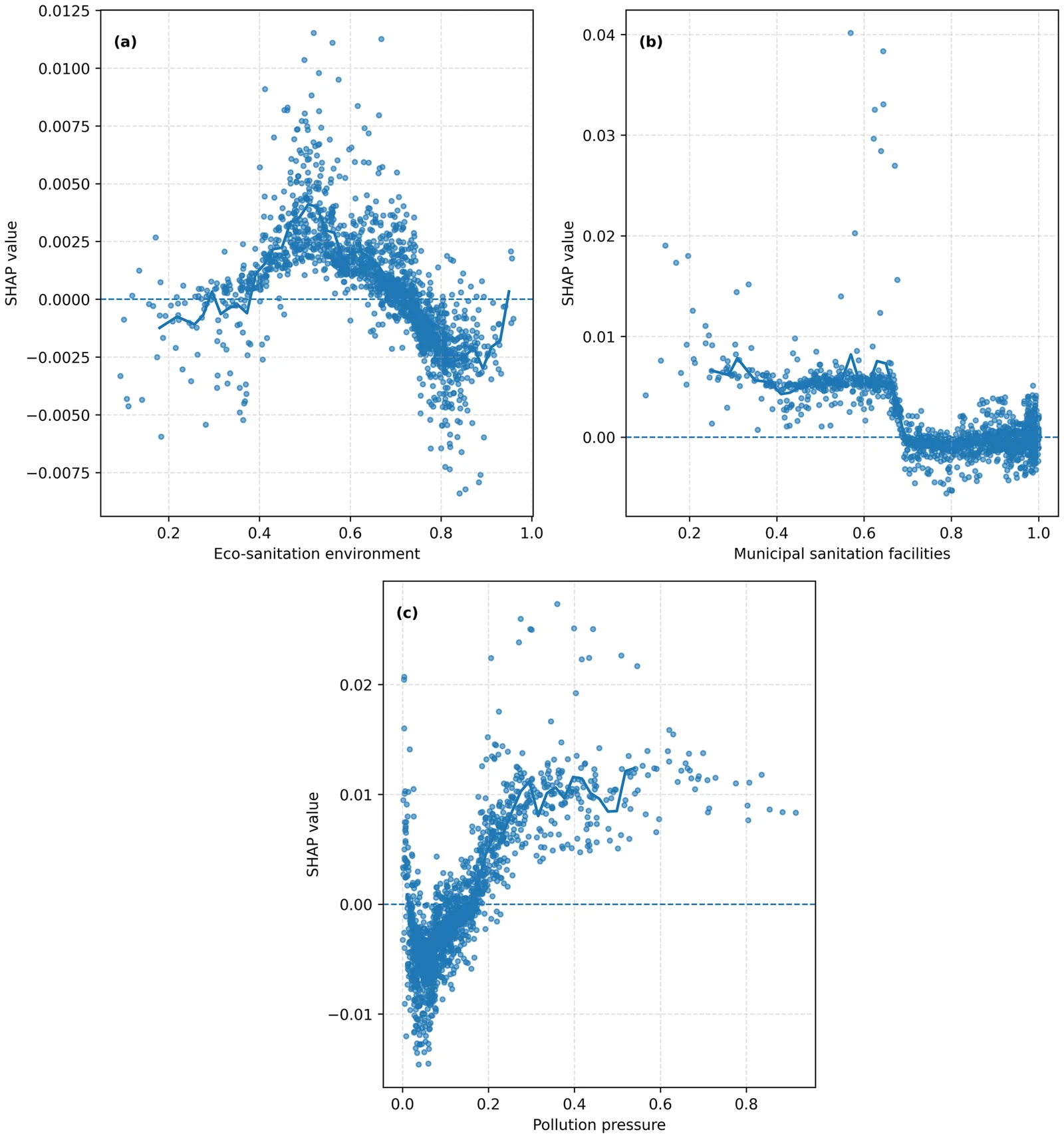
Nonlinear dependence between key variables and health service supply capacity

Figure 18 further shows the quantile dependence relationships between the eco-sanitation environment, municipal sanitation facilities, pollution pressure, and health service supply capacity. The marginal relationships of the eco-sanitation environment and municipal sanitation facilities tend to flatten at high values, while pollution pressure shows a clear nonlinear increasing relationship with health service supply capacity. This is consistent with the earlier interpretation of pollution pressure as a resource-allocation response and also suggests that average effects in conventional linear models may conceal differences across cities at different stages of development.

**Fig. 19:**
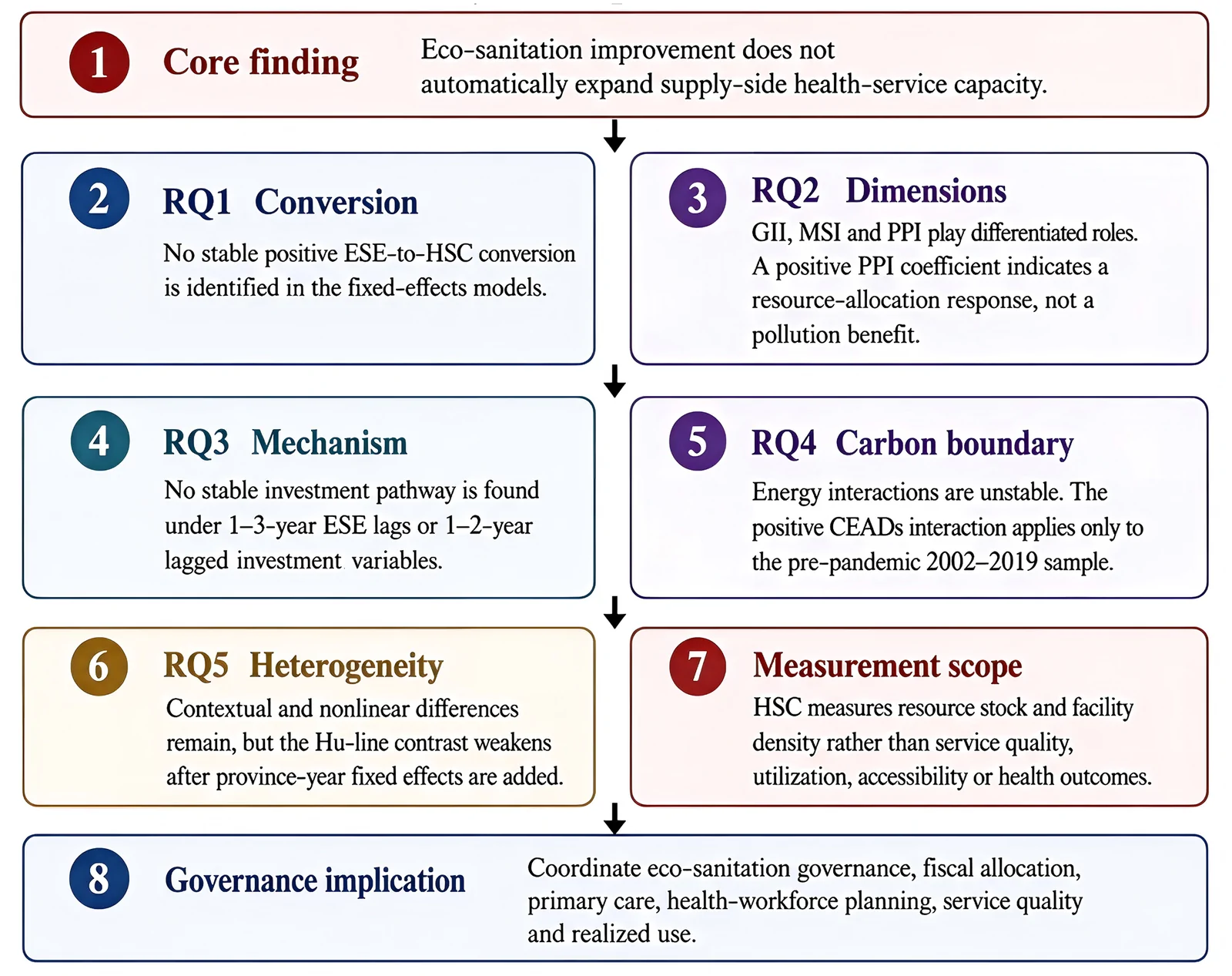
Summary of empirical evidence for the research questions

Finally, Figure 19 summarizes the evidence for the five research questions. The results indicate no stable positive conversion from ESE to HSC and differentiated roles across ESE dimensions. The positive PPI coefficient represents a supply response under pollution pressure rather than a pollution benefit. The investment pathway remains unsupported under one- to three-year timing structures. The positive carbon interaction is limited to the CEADs-matched pre-2020 period. City-context heterogeneity and nonlinear boundaries remain evident, but the Hu-line subgroup contrast is attenuated after province-year policies are absorbed.

## Discussion

### From positive-effect assumptions to mismatch mechanism identification

The core finding of this study is not that eco-sanitation improvement has a stable positive effect on health service supply capacity, but that there is a clear conversion mismatch between the two. During 2002–2024, the eco-sanitation environment, green infrastructure, and municipal sanitation facilities of Chinese cities improved continuously, and pollution pressure generally declined; however, health service supply capacity improved more slowly and relied mainly on the expansion of beds and licensed physicians rather than on a synchronous increase in the number of medical institutions. The two-way fixed effects model further shows that the lagged eco-sanitation environment does not exhibit a stable positive average effect. This finding suggests that although eco-sanitation construction can improve the foundation for public health, its conversion into health service supply capacity is jointly constrained by healthcare resource allocation, interdepartmental coordination, population demand, primary service networks, and the stage of urban development.

The theoretical implication of this study is therefore to move eco-sanitation research beyond the single question of whether it produces positive health effects and toward a health service governance perspective that asks whether environmental improvement can be converted into health service supply capacity. Eco-sanitation improvement can reduce health risks, improve urban livability, and strengthen the foundations of public health, but it does not automatically expand beds, physicians, primary service outlets, or medical institutions. Health service supply capacity is more directly affected by population size, fiscal capacity, service network planning, health workforce allocation, and the service functions of cities.

Accordingly, the negative baseline coefficient is not a “better environment–poorer healthcare” effect. It indicates that, within the same city and conditional on the included controls, years following faster improvement in the measured eco-sanitation infrastructure were not accompanied by proportional growth in supply-side medical resources. Such a pattern can arise from different adjustment cycles, segmented budgets, saturation of basic municipal facilities, or the concentration of new medical resources in population-inflow and regional-center cities. Because the coefficient is small, specification-sensitive, and derived from an observational design, it should be read as a mismatch signal rather than a causal welfare effect.

### Temporal, spatial, and institutional mismatches

The baseline regressions, dimension-specific models, and mechanism tests jointly indicate temporal, spatial, and institutional mismatches. Green space and municipal sanitation facilities are commonly implemented through construction and environmental-governance systems, whereas hospital siting, physician training, staffing approvals, and health budgets operate over longer and institutionally distinct cycles. The reviewer-motivated timing tests show that allowing two- and three-year ESE lags does not activate a stable investment pathway, and one- and two-year lagged investment variables likewise remain mostly unrelated to HSC. This evidence suggests that temporal delay alone does not explain the missing transmission chain.

The extended mechanism evidence does not support a stable ESE → urban construction investment → HSC chain. The two-year ESE specification identifies only a negative wastewater-investment association, and the three-year specification weakens this relationship to the 10% level; none of the corresponding investment variables significantly predicts HSC. One-year lagged landscaping investment is weakly positive, but the effect disappears at two years and is not reproduced by other mechanisms. Accordingly, the null mechanism conclusion is robust to plausible multi-year lags, although missing values and annual volatility still reduce power. The findings imply that eco-sanitation projects are unlikely to translate automatically into health-resource expansion unless medical service layouts, primary care networks, staffing decisions, and fiscal allocation are jointly planned.

### Pollution pressure, energy-carbon conditions, and resource-allocation responses

The positive PPI coefficient should not be interpreted as evidence that pollution has health benefits. PPI captures emissions intensity and PM₂.₅ pressure, while HSC captures supply-side resource counts. A positive within-city association is therefore consistent with a compensatory or demand-induced response: pollution may raise latent healthcare demand, and industrial or populous cities may simultaneously possess greater fiscal and organizational capacity to expand beds, physicians, and institutions. This interpretation does not imply improved quality of care, utilization, health outcomes, or a causal benefit of pollution.

The energy and carbon results also reflect development-stage and sample-boundary differences. Pollution-pressure and energy interactions remain unstable, whereas the CEADs interaction is positive in the available matched sample. Exact-sample comparisons confirm a positive interaction through 2019, but the CEADs series does not observe COVID-19 or subsequent changes in healthcare demand, public-health spending, hospital capacity, or intergovernmental allocation. The carbon result is therefore a pre-pandemic conditional association and should not be generalized to 2020–2024. Its positive sign is interpreted as the resource-allocation capacity associated with high-emission cities, not as a benefit of carbon emissions.

### Urban heterogeneity and nonlinear boundaries

The primary heterogeneity models show differences by region, GDP level, and population size. For the Hu Huanyong line, however, the southeastern coefficient loses conventional significance after province-year fixed effects are introduced, while the northwestern coefficient remains imprecise and insignificant. This indicates that time-varying provincial policy environments account for part of the initial geographic contrast. The Hu line should therefore be treated as a descriptive urban-system grouping rather than as an independently identified boundary effect. The small northwestern sample also limits precision.

Interpretable machine learning further supports this interpretation. The random forest model shows good predictive performance. SHAP results indicate that per capita GDP, population size, the tertiary industry share, and financial development are the main variables explaining differences in health service supply capacity. Eco-sanitation variables have relatively lower importance, but green infrastructure and pollution pressure still contribute to model explanations. SHAP dependence relationships show that the marginal effects of the eco-sanitation environment and municipal sanitation facilities flatten at high values, while pollution pressure has a nonlinear relationship with health service supply capacity. Thus, the evidence supports RQ5 regarding urban heterogeneity and nonlinear boundaries.

### Policy implications and limitations

The results indicate that healthy city development cannot rely solely on eco-sanitation improvement; ecological governance, municipal infrastructure, and health-resource allocation should be coordinated. These implications apply specifically to supply-side resource availability and service-network density. More beds, physicians, or institutions do not necessarily imply better clinical quality, effective coverage, equitable accessibility, efficient utilization, continuity of care, or improved patient outcomes. Policy monitoring should therefore pair supply indicators with utilization rates, service quality, travel-time accessibility, primary-care continuity, patient-reported experience, and outcome measures.

Policy strategies should be differentiated by city type and linked to health-service governance. Eastern, densely populated, and high-GDP cities should optimize the spatial matching of ecological spaces, primary-care outlets, and higher-level medical resources. Central and western, smaller, and fiscally weaker cities should fill basic supply gaps while improving eco-sanitation infrastructure. High-pollution cities should jointly evaluate latent health demand, workforce allocation, primary-care capacity, and pollution-control performance. Because the carbon moderation evidence ends in 2019, it should not be used alone to prescribe post-pandemic resource allocation without updated carbon and health-service data. Across all city types, performance frameworks should distinguish resource quantity from service quality and realized use.

This study has several limitations. First, HSC is based on beds, physicians, and hospitals/health centers per 10,000 persons. It measures supply-side resource stock and network density but does not adjust for quality, utilization, accessibility, efficiency, case mix, unmet need, patient experience, or health outcomes; policy conclusions must remain within this operational scope. Second, city-level data cannot capture within-city inequalities across neighborhoods and population groups. Third, the CEADs inventory ends in 2019, so carbon moderation is pre-pandemic and cannot capture post-2019 health-resource restructuring. Fourth, although longer lags were tested, missing values and volatility in investment variables may still limit power to detect weak delayed mechanisms. Fifth, province-year fixed effects reduce confounding in the Hu-line analysis, but the northwestern subgroup remains small and the line itself is not an exogenous treatment. Sixth, the design is observational and does not support strict causal claims. Seventh, stable city-center coordinates, adjacency matrices, and commuting-flow matrices are unavailable, leaving possible spatial spillovers untested. Future research should combine claims or utilization data, quality indicators, geocoded facilities, resident surveys, updated post-2019 carbon inventories, spatial weights, and quasi-experimental designs.

The potential consequences of unmodeled spatial dependence require further clarification. City fixed effects absorb time-invariant spatial characteristics, year fixed effects absorb common national shocks, and the control variables and province-year fixed-effects sensitivity analysis absorb part of the observed regional and policy heterogeneity. These adjustments reduce, but do not eliminate, spatial dependence. If spatial correlation is confined to the regression disturbance and the regressors remain conditionally exogenous, the principal concern is inaccurate or inefficient inference rather than systematic bias in β. By contrast, β may be biased if omitted spatially lagged HSC, neighboring-city eco-sanitation investment, or other spatially correlated shocks are correlated with ESE. City-clustered standard errors do not resolve that form of omitted-variable bias. Because a theoretically defensible and temporally stable spatial weights matrix cannot be constructed from the available data, the study cannot determine which case dominates. The reported β is therefore treated as a conditional within-city association, not a spatially isolated or causal effect, and possible cross-city spillovers remain a substantive source of uncertainty.

## Conclusion

This study does not assume that eco-sanitation improvement is a sufficient condition for enhancing health service supply capacity. Instead, using panel data for Chinese prefecture-level and above cities from 2002 to 2024, it examines whether eco-sanitation improvement can be converted into health service supply capacity and identifies the mismatch mechanisms and boundary conditions in this process. The study constructs indices of urban health service supply capacity, eco-sanitation environment, green infrastructure, municipal sanitation facilities, and pollution pressure, and combines two-way fixed effects models, mechanism tests, moderation analysis, heterogeneity analysis, and interpretable machine learning.

The results show that the eco-sanitation environment, green infrastructure, and municipal sanitation facilities in Chinese cities improved continuously, and pollution pressure generally declined; however, health service supply capacity improved more slowly and was mainly driven by the expansion of beds and licensed physicians rather than by a synchronous increase in the number of medical institutions. The baseline regressions show that the lagged eco-sanitation environment does not exhibit a stable positive average effect on health service supply capacity, indicating that eco-sanitation improvement does not automatically translate into stronger healthcare resource supply capacity. Instead, temporal, spatial, and institutional mismatches exist between the two. This negative association does not mean that ecological improvement reduces healthcare capacity; it indicates that growth in the measured eco-sanitation infrastructure and growth in supply-side medical resources were not synchronized within cities. Spatial spillovers remain unmodeled, so the coefficient should be interpreted as a conditional association rather than a spatially independent causal effect.

Dimension-specific results show different roles for green infrastructure, municipal sanitation facilities, and pollution pressure. The positive PPI coefficient reflects a likely healthcare resource-allocation response under pollution pressure and must not be interpreted as a pollution benefit. The absence of a stable construction-investment pathway persists under two- and three-year ESE lags and one- and two-year lagged investment variables. The positive CEADs interaction is limited to matched observations through 2019 and therefore provides pre-pandemic evidence only.

Heterogeneity and interpretable machine learning reveal city-context differences and nonlinear boundaries. Regional, GDP, and population patterns remain evident. The initial southeastern Hu-line significance is attenuated after province-year fixed effects are included, so the line should not be treated as an independently identified geographic effect. SHAP results indicate that economic development, population size, industrial structure, and financial development explain more variation in HSC than eco-sanitation variables.

The study extends environmental-health research from individual outcomes to the conversion of urban environmental improvement into supply-side health resources, integrates eco-sanitation and energy-carbon conditions, and combines panel econometrics with interpretable machine learning. Its policy contribution is limited to the availability and distribution of health-service resources; stronger supply indicators should not be equated with higher service quality, utilization, accessibility, or overall health-system performance.

## Electronic supplementary material

Below is the link to the electronic supplementary material.

Supplementary material 1

## Data Availability

The processed dataset and supporting materials used in this study are publicly available in the Mendeley Data repository, Version 1, at https://doi.org/10.17632/hw4j6xv99v.1 [62]. The repository contains the analytical panel data, variable definitions, index weights, model specifications, statistical results, and machine-learning outputs. The original source data were obtained from the official statistical sources described in the Methods section and the source manifest included in the repository.

## Abbreviations

CEADs: Carbon Emission Accounts and Datasets
CO₂: Carbon dioxide
ESE: Eco-sanitation environment index
FE: Fixed effects
GDP: Gross domestic product
GII: Green infrastructure index
GIS: Geographic information system
HSC: Health service supply capacity index
LIME: Local Interpretable Model-Agnostic Explanations
MAE: Mean absolute error
MSI: Municipal sanitation facility index
N: Number of observations
NOₓ: Nitrogen oxides
PCA: Principal component analysis
PGDP: Per capita gross domestic product
PM₂.₅: Particulate matter with an aerodynamic diameter of 2.5 μm or less
PPI: Pollution pressure index
R&D: Research and development
R^2^: Coefficient of determination
RMSE: Root mean squared error
RQ: Research question
SD: Standard deviation
SE: Standard error
SHAP: SHapley Additive exPlanations
SO₂: Sulfur dioxide
Tce: Tonne of coal equivalent
VIF: Variance inflation factor
